# Tumor microenvironment conversion through intelligent nanomedicine: a paradigm shift in overcoming chemoresistance

**DOI:** 10.3389/fonc.2026.1812990

**Published:** 2026-06-17

**Authors:** Maliheh Hasannia, Mahdi Abounoori, Fatemeh Mahmoudian, Maryam Shirzad, Abbas Rahdar, Selamu Duguna, Sadanand Pandey

**Affiliations:** 1 Cancer Research Center, Semnan University of Medical Sciences, Semnan, Iran; 2 Social Determinants of Health Research Center, Semnan University of Medical Sciences, Semnan, Iran; 3 Nanotechnology Research Center, Pharmaceutical Technology Institute, Mashhad University of Medical Sciences, Mashhad, Iran; 4 Department of Physics, University of Zabol, Zabol, Iran; 5 Faculty of Applied Sciences and Biotechnology, Shoolini University, Solan, Himachal Pradesh, India; 6 Department of Chemistry, College of Natural Science, Yeungnam University, Gyeongsan, Republic of Korea

**Keywords:** cancer nanotechnology, chemoresistance, intelligent nanomedicine, pH-responsive nanocarriers, targeted drug delivery, tumor microenvironment (TME)

## Abstract

Chemoresistance remains a major challenge in effective cancer management, significantly limiting the therapeutic efficacy of conventional chemotherapy and contributing to tumor recurrence, metastasis, and poor clinical outcomes. Increasing evidence highlights the pivotal role of the tumor microenvironment (TME) in driving therapeutic resistance through complex biological and physicochemical barriers, including hypoxia, acidic pH, abnormal vasculature, elevated interstitial fluid pressure, immune suppression, and dense extracellular matrix deposition. These TME-associated factors collectively impair drug penetration, promote tumor survival, and reduce chemosensitivity. Consequently, contemporary anticancer strategies are shifting from direct tumor cell eradication toward dynamic modulation and conversion of the TME. In this context, intelligent nanomedicine has emerged as a transformative platform capable of precisely responding to endogenous and exogenous stimuli such as pH, redox gradients, hypoxia, enzymes, light, and magnetic fields for site-specific drug delivery and controlled therapeutic activation. Stimuli-responsive nanocarriers not only enhance intratumoral drug accumulation and penetration but also actively remodel the TME through immunomodulation, vascular normalization, extracellular matrix degradation, photodynamic and photothermal effects, and catalytic reactive oxygen species generation. Such multifunctional nanosystems can effectively reverse chemoresistance by reprogramming the tumor milieu into a therapeutically responsive state. This review comprehensively discusses recent advances in intelligent nanomedicine-mediated TME conversion strategies, their mechanistic role in overcoming chemoresistance, and their translational potential in precision oncology. Collectively, TME-targeted intelligent nanomedicine represents a paradigm shift in cancer therapeutics, offering new opportunities for improving therapeutic efficacy while minimizing systemic toxicity.

## 1 Introduction

### 1.1 Tumor microenvironment: a major barrier in cancer treatment

The TME is made up of a wide array of cell types, both neoplastic and non-neoplastic, and non-cellular elements. Among these non-neoplastic stroma cells are CAFs, endothelial cells, and pericytes that assist in tumor angiogenesis and immune cells involved in the disease (1). Tumor-associated characteristics develop within neoplastic and stromal cells due to changes in their morphology, metabolism, and genetics during tumorigenesis. The ECM and the secreted extracellular molecules can influence the development of the tumor through autocrine and paracrine processes, together with the cellular component. Recent studies show that stromal cells play an important role in tumor formation, growth, and metastasis by (a) secreting signaling molecules, (b) forming and remodeling the extracellular matrix, and (c) regulating the angiogenesis of cancer (2). It is necessary to understand the importance of stroma cells in the process of tumorigenesis by knowing the varied populations of Tumor stroma and its relations to cancer cells. Recent research has shown the important contribution of stromal cells in cancer initiation and progression. There have been many studies published on the biology of stromal cells, highlighting their roles and contributions of other components in cancer progression and metastasis (3–7).

For the rapid growth of cancer cells, they require a certain amount of oxygen and nutrition, which can be attained through neovasculature generation by a biological phenomenon known as angiogenesis. The formation of tumor vasculature is quite varied from normal tissues since the rate of cell division in endothelial cells is slow when compared with tumor cells; hence, tumor vasculature fails to match the division of tumor cells (8). This makes the morphology of the generated neovasculature abnormal, comprising dilated, tortuous, irregular, and large fenestration. The basement membrane and endothelial lining of tumor vasculature may be deficient, together with pericyte, smooth muscles, and pharmacological receptor absence. Fluid and solid stresses together with an increase in the permeability of vessels are some of the mechanical factors that affect the slow and non-uniform flow of blood seen in Tumors. Solid stress brought about by tumor growth and dense extracellular matrix can pressurize the blood vessels and make it difficult for them to carry blood hence resulting in the leakage of plasma into the tumor cells. This causes high vascular permeability leading to high interstitial fluid pressure making it difficult for the transport of drug molecules because of the pressure gradient caused by the abnormal vasculature of the tumor (9).

IFP is another factor that can impede the movement of NPs in the intratumoral region. In healthy tissue, the normal IFP is usually between 0 and 3 mmHg. In solid tumors, however, the IFP rises to 5 to 40 mmHg; while in the desmoplastic type of pancreatic cancer, the IFP may rise up to 75 to 130 mmHg (10). In solid tumors, interstitial fluid pressure (IFP) is elevated due to several factors, including hyperperfusion of abnormal tumor vasculature, poor lymphatic drainage, and the high density of the extracellular matrix (ECM). Increased fluid uptake and serum protein absorption in solid tumors increase fluid permeability through defective blood vessels (11).

Furthermore, stromal burden increases due to the increased proliferation of Tumor cells, formation of dense elastin and collagen matrixes, and contractility of fibroblasts. The importance of IFP at the site around the Tumor margin attains a normal value, thus creating a sharp pressure gradient. Transport of NPS through the tumor interstitium is highly restricted since transport of drugs takes place by diffusion mechanism, particularly in case of large particles (12). Higher IFP can help in the return of the drugs into the circulatory system. At the same time, IFP at the site of the tumor microenvironment helps interstitial fluid to migrate from the tumor margin to other tissues, along with carrying out NPS, growth factors, and cells (13).

The extracellular matrix comprises a cross-linked network of collagen proteins, glycoproteins, proteoglycans, elastin fibers, and hyaluronan. The components, growth factors, and enzymes found in the extracellular matrix play a vital role in regulating cell proliferation, differentiation, survival, and homeostasis. The nature and structure of the extracellular matrix in normal tissues differ from that in cancerous tissues. The standard extracellular matrix at the cellular level occurs within the basement membrane and interstitial matrix. The extracellular matrix of the basement membrane is made up of laminins, fibronectin, and collagen type IV, characterized by high density and lower porosity. The interstitial matrix comprises collagen type I, glycoproteins, and proteoglycans, increasing tissue tensile strength (14). Tumor stroma entails modified extracellular matrix associated with stromal cells, including fibroblasts, pericytes, endothelial cells, and immune cells. Cancer-associated fibroblasts are essential for the production of the extracellular matrix of the tumor. Cancer-associated fibroblasts increase the amounts of collagens and proteoglycans, which together with the lysyl oxidase secreted by the tumor stroma may lead to the development of a dense ECM (15).

ECM increased stiffness enhances Tumor survival and proliferation via activation of integrin signaling pathways. Tumor associated cancer fibroblasts synthesize extracellular enzymes like plasmin, disintegrins, and matrix metalloproteinases (MMPs), which can lead to degradation of ECM and alteration of ECM structure, enhancing the invasiveness of cancers. The tumor associated ECM can lead to alterations in biochemical composition of ECM. ECM rigidity in breast cancer stroma might be about tenfold more than that in normal breast tissue (16). LOX plays an important role in maintaining the ECM stiffness. LOX production may occur due to cross-linking between collagen fibers and ECM components. Initially, the TGF-β1 signaling pathway initiates LOX production. It may also occur due to secretion by the cancer cells under conditions of hypoxia at late stages of tumor development. Increased density of ECM along with proliferation of neoplastic cells may cause condensation of tumor vessels, thus inhibiting the transport of nanoparticles through the vascular walls. Polymer-based components of ECM such as hyaluronic acid and collagen molecules hinder the transport of charged nanoparticles (17).

Compared to conventional chemotherapeutic drugs, studies have proven that chemotherapeutic agents formulated in NPs have superior pharmacokinetics and less toxicity effects. Nevertheless, due to physiological barriers and variation in the EPR effect in solid tumors, which limits even distribution and infiltration of nanomeds, nanomeds often exhibit lower therapeutic benefits in comparison with their parent compounds (18, 19). A variety of nanoparticulate formulations approved by the FDA, including paclitaxel formulated with albumin (Abraxane), daunorubicin formulated with citrate liposomes (DaunoXome), and doxorubicin formulated with liposomes (Doxil/Caelyx), exhibit lower toxicity than conventional chemotherapy drugs, contributing to only a small increment in survival rates by 22%. The tumor microenvironment as well as the physicochemical properties of NPs, play critical roles in their ability to penetrate tumors (20).

Abnormality in the vascular structure of tumors, which comprises high permeability and lacks lymphatic drainage systems, coupled with the presence of high extracellular matrix density and high interstitial fluid pressure due to fast growth of tumor cells, represents biological barriers that reduce the diffusion capacity of nanoparticles and hinder their delivery to the target tissue. Some of the physical properties of the nanoparticles, such as size, surface charge, and shape, influence the efficacy of nanomedicine in penetrating tumor masses. Nanomedicines’ performance may be compromised due to the existence of tumor heterogeneity and abnormal tumor microenvironment in addition to nanoparticle properties (21).

Heterogeneity of tumor leads to variable cut-off size pores in tumor vasculature, preventing the selective extravasation of larger nanoparticles due to the enhanced retention and permeability effects. For successful transport of nanoparticles, the EPR effect alone is insufficient. The EPR effect for various tumors depends on the density of densely packed vessels and hypoxia. Several factors affect the EPR effect including:(i) functional lymphatic vasculature and angiogenesis; (ii) proliferative activity of tumor cells adjacent to tumor vessels and mechanical stress generated by tumor hyperplasia and tumor stroma; (iii) co-mediators affecting blood flow and stroma; and (iv) mononuclear phagocytic activity (22).

As a result, when NPs reach the tumor microenvironment, they face physiological barriers, which impede nanomedicine delivery. Consequently, it is imperative to design nanomedicine that considers how it interacts with these barriers. Recently, drug delivery system design has taken into consideration the development of multistage delivery systems capable of modifying the size of NPs in response to external and internal stimuli, thereby allowing NPs to overcome challenges in the tumor microenvironment and penetrate into tumors. There are two general approaches to increasing tumor penetration of NPs, namely, (1) the improvement of physicochemical properties of NPs; and (2) the modification of the tumor microenvironment. This article examines strategies for designing multifunctional NPs for tumor penetration (23).

### 1.2 Chemoresistance in cancer: mechanisms and clinical impact

Both Solid and hematologic types of tumors can be treated by chemotherapeutic drugs. The chemotherapeutic treatment acts on rapidly proliferating tumor cells, but at the same time, it impacts normally proliferating cells, hence making them resistant to medication (24). One of the challenges in using chemotherapy in treating cancers is that cancer cells become chemoresistant, making it one of the key factors responsible for cancer deaths. Genes and epigenetics affect the uptake, processing, and drug removal mechanisms and ultimately result in chemoresistance. As documented by reports, most of the cancer deaths are due to the failure of chemotherapy or targeted therapies, about 90%. During chemotherapies, MDR results from genetic and epigenetic modifications (25). Understanding the underlying mechanisms of drug resistance is essential for the development of effective cancer therapies. Drug resistance in cancer cells arises due to multiple factors, including tumor heterogeneity, overexpression of efflux pumps, genetic mutations, and alterations in cellular signaling pathways. To overcome these challenges, several advanced therapeutic strategies have been developed, such as targeted therapy, immunotherapy, combinational therapy, and other molecular-based approaches, which collectively aim to enhance treatment efficacy and minimize resistance in cancer cells (26).

The reason for failure of most chemotherapeutic interventions is attributed to multidrug resistance (MDR). This resistance is regarded as either intrinsic or acquired (27). Malignancies that are intrinsically resistant are not affected by chemotherapeutics from the outset, thus rendering therapy inefficient. In acquired resistance, the cancerous cells initially react to therapy but eventually become resistant to other drugs too (28). In any case, these forms of resistance interfere with effective therapy. About 90% of ovarian cancer fatalities can be attributed to drug resistance (29). Drug resistance can occur with both old and new chemotherapeutic drugs (30).

The progression of cancers is associated with changes in the TME, such as ECM remodeling, ECM stiffness, vascularization, hypoxia, and paracrine signaling. Cancer cells may develop resistance to chemotherapeutic drugs. Signaling pathways activated by ECM components influence genetic and protein functioning, thereby increasing the likelihood of drug resistance. Additionally, the ECM affects the activity of cancer-related stromal cells, causing further changes in the microenvironment, resulting in resistance development. Previously, attention has been paid exclusively to intrinsic, Tumor cell-mediated chemoresistance. Recent findings suggest an integral part played by the TME in inducing therapy resistance and Tumor development. In oncology studies, the Tumor microenvironment plays an equal role to the Tumor cells (31).

### 1.3 Implications for clinical nanocarrier selection

There is a significant gap between the remarkable success of nanocarriers in experimental studies and their limited impact in clinical oncology. It indicates a clear disjunction between the parameters, according to which nanocarriers are validated in laboratories and the conditions of the disease they have to overcome in reality. Therefore, improving the criteria for selecting suitable nanocarriers for clinical implementation has become an urgent priority for researchers (32).

First and foremost, it concerns the use of appropriate models in order to test nanocarriers. Subcutaneous xenografts, traditionally used in preclinical studies, are far from reflecting human tumor biology because of the lack of heterogeneity of architecture, stroma density, vascular characteristics, and immunological diversity. In other words, nanocarriers which have been successfully tested in such simplified systems often appear to be inadequate in reality. For this reason, the choice criteria of nanocarriers for further development should give preference to those which show efficient behavior in advanced models, i.e., orthotopic tumors, patient-derived xenografts (PDX), and humanized mice. Such models are capable of reflecting critical aspects of pathologies, such as immune dynamics and tumor-stroma interaction (33, 34). Furthermore, the irregular geometry of tumors and the heterogeneity of perfusion seen in human cancer cases pose major hurdles to the delivery of nanoparticles into tissues. The use of nanocarriers which have been optimized in stringent pre-clinical systems that can mimic such physiological conditions provides more compelling justification for clinical testing (35).

In addition to biological relevance, manufacturability is also an important factor in determining whether a nanocarrier is suitable for clinical trials. Highly complex structures, multicomponent systems, or difficult-to-control synthesis methods can create challenges in large-scale production and quality control. Therefore, formulations with well-defined chemical compositions, simple synthesis procedures, and GMP-compliant manufacturing processes are generally preferred (36).

Regulatory agencies require a comprehensive physicochemical characterization of nanoparticles, including parameters such as size distribution, surface charge, stability, and batch-to-batch uniformity. Nanocarriers that are not reproducible will not move along the regulatory pathway irrespective of their preclinical efficiency. Therefore, the manufacturability of nanocarriers should be viewed as another important selection criterion rather than an afterthought (37). Toxicology and pharmacokinetic predictability are two other crucial factors contributing to successful clinical implementation. Many nanocarriers prove to have a good safety profile in rodents; however, this does not reflect their tendency to accumulate in the liver, spleen, or mononuclear phagocyte system over a more extended period compared to humans. Clinical nanocarriers must show evidence of biodegradability, little off-target retention, and reduced risk of adverse immune responses (38).

The biological target selected for activating or accumulating nanocarriers is another important factor determining the potential for translational success. Hypoxia, acidity, and enzymatic conditions may serve as effective triggers for activating nanocarriers in animal models; however, their efficacy is inconsistent in human tumors because of substantial differences in their characteristics. On the contrary, nanocarriers exploiting known biomarkers of the disease, such as receptor overexpression and molecular signature, are more reliable (39).

Using targets that are measurable and constant across patient populations enables clinicians to stratify patients before treatment. This approach enables the use of nanocarriers in patients who are more likely to respond positively to the treatment, thereby enhancing therapeutic outcomes and improving treatment efficiency. Nanocarrier design which has a combination of diagnostic and therapeutic capability may prove to be useful in improving the translation of nanocarriers into clinic. The use of such nanocarriers which can image and deliver drug enables real time imaging of biodistribution and drug delivery profiles (40).

Imaging nanocarrier activity *in vivo* is beneficial in that it will help the clinician make informed decisions regarding the treatment regimen, and also helps in dose optimization. In other words, the activity is important for making appropriate treatment decisions and developing evidence-based clinical application (41).

It is also important for candidate nanocarriers to be assessed for their integration within standard clinical practice. Delivery devices which are special and unique ways of administering drugs, for example, are less likely to be integrated into clinic. On the other hand, nanocarriers which are easy to integrate with existing infusion and localized delivery protocols are likely to find favor in clinical practice (42). The economic feasibility of a potential nanocarrier system will determine whether its development is economically realistic enough to allow its implementation in the clinic. The expensive manufacturing process, complex purification procedures, or the use of exotic materials might make an otherwise efficient nanocarrier system unfeasible. The issue of cost efficient scalability should thus be addressed from the early stages of design (37).

An additional aspect that should be taken into account when designing a clinically effective nanocarrier system is the reproducibility of their behavior in the clinic among patients exhibiting biological differences. Heterogeneity in tumor vasculature, density of stroma and immune cells, and metabolic states can make some nanocarriers less efficient than others in diverse biological settings (43).

Highlighting practical concerns of clinical relevance will also direct researchers towards designing nanocarriers that will be compatible with current clinical practices in terms of timing and compliance. Taken together, these points emphasize that there is an urgent requirement to change the paradigm in choosing the nanocarriers for clinical testing. Rather than selecting the most advanced or sophisticated techniques, it is essential to concentrate on the carriers that have the highest probability of being clinically effective, approved by regulatory agencies, and scalable (40).

### 1.4 Nanomedicine in overcoming TME barriers

The delivery of medications to tumors via nanoparticles is an area that has attracted significant attention in recent years. It is extremely difficult for drugs to be administered into the Tumor due to the complex nature of the tumor microenvironment (44). In this regard, the challenges associated with the tumor microenvironment have been summarized. These challenges include suppression of the immune system, hypoxia, low pH, dysregulation of enzymes, rigid extracellular matrix, dysregulated blood vessel structure, and dysregulated metabolism. Different approaches have been proposed to overcome these challenges; these include tumor microenvironment modulation, active targeting, biomimetic strategies, and tumor microenvironment-responsive drug delivery systems (21).

As discussed above in the previous section, there are still some challenges to be addressed even though there have been great advancements. The discussion entailed the current status of TME, advances in drug delivery systems, and aspects that require more emphasis. There are increasing reports showing that tumor heterogeneity plays a vital role in drug delivery via nanocarriers. This comprises attributes derived from cancer cells and their interactions within the tumor microenvironment, such as aberrant vasculature, stiff extracellular matrix, hypoxia, acidic pH, high glutathione (GSH) concentration, reactive oxygen species (ROS), and immunological inhibition (45). Inclusion of an overview of the dynamic Tumor environment will enhance our comprehension of the upcoming drug delivery strategy. The challenging barriers of the Tumor microenvironment largely obstruct drug delivery into the Tumor. According to recent studies, Tumor heterogeneity greatly obstructs drug delivery using nanocarriers. Factors such as aberrant vasculature, stiff extracellular matrix, hypoxia, acidic pH, high GSH concentration, and immunological inhibition are derived from cancer cells and their interaction within the TME (45).

The field of research involving nanoparticles for targeted tumor delivery is evolving. Targeted drug delivery to tumors is hindered by many barriers caused by the tumor microenvironment. Some of the barriers in TME are detailed in this study and include immune-suppressing conditions, hypoxia, acid pH, abnormal enzyme concentration, rigid extracellular matrix, abnormal vasculature, and modified metabolic pathways. Various methods have been developed to overcome these barriers to enhance nanoparticle penetration, cell internalization, and drug release through biomimetic approaches, ligand-based active targeting, tumor microenvironment engineering, and tumor microenvironment responsive drug delivery. Research indicates that the heterogeneous nature of tumors greatly affects drug delivery via nanocarriers. Some of the barriers include abnormal vasculature, rigid extracellular matrix, hypoxia, acid pH, high glutathione and reactive oxygen species concentration, and immunosuppression. These barriers arise from cancer cells and their interactions with the tumor microenvironment (46, 47).

## 2 Strategies for designing nanocarriers aimed at the tumor microenvironment

### 2.1 The EPR phenomenon is one of the pivotal concepts of the tumor-targeting therapy using nanomedicine, whereby there is a natural propensity of the nanoparticles to be passively accumulated in cancerous cells

The reason behind this stems from the special physiological nature of tumor vessel walls that differ greatly from normal tissue vessel walls (48). The walls of normal blood vessels are impermeable compared to tumor vessel walls, whose structure is defective and features wide gaps between the endothelial cells (between 100 and 800 nm), discontinuous basement membrane, and absence of smooth muscle cells. This permits macromolecular particles and nanoparticles having dimensions between 10 and 200 nm to move selectively into the Tumor Interstitium (49).

There is an insufficient or poorly functioning lymphatic drainage system in Tumors. Lymphatic systems can drain interstitial fluid and macromolecules out from healthy tissues. Due to the inability of the lymphatic system to drain macromolecules out, Tumors retain extravasated nanocarriers; hence, they cannot rapidly washout and accumulate more in Tumors. The combination of high permeability and poor lymphatic drainage enhances the action of the EPR effect leading to the accumulation of more drugs by Tumors compared to healthy tissues, reducing systemic toxicity (50).

The clinical application of the EPR effect has been highly advantageous, especially in drug formulations such as DOXIL^®^, a PEGylated liposomal form of doxorubicin that has been approved by the FDA. DOXIL^®^ utilizes the EPR effect for effective accumulation by Tumors, resulting in more efficacy and reduced cardiotoxicity when compared to conventional doxorubicin (51, 52). It is well-known that the efficacy of the therapeutic application of EPR can vary from one type of tumor to another and also between different patients, influenced by such parameters as the size of the Tumor, its vascular density, and interstitial fluid pressure. Recently there have been attempts to find ways of improving the EPR phenomenon for better nanocarrier distribution (52).

### 2.2 Active targeting of tumor -specific markers

The use of active targeting approaches attempts to overcome some of the limitations of passive targeting and increase treatment precision through functionalizing nanocarriers with specific ligands that interact with overexpressed biomarkers within the TME. Such an approach will improve the effects of the EPR effect by allowing for the binding of the targeted nanocarriers and subsequent internalization within the target cell leading to higher intracellular drug concentrations (53). Antigens, proteins, or other molecules found on the surface of cancer cells and/or tumor-related endothelial cells can be used as ligands bound onto selected areas of nanocarriers to target specific receptors or proteins (54). Folate, transferrin, and HER2 are examples of such biomarkers (55).

Peptides containing the RGD sequence, which have high expression on angiogenic endothelial cells, are commonly used as targeting agents against αvβ3 integrins, providing an effective way to cut off the blood supply of the tumor (56). New developments show the therapeutic promise of this method through its transition from preclinical studies to human subjects (57, 58). Recent clinical evidence suggests that phase I clinical trials involving nanoparticles with MUC1 targeting and paclitaxel as payloads were effective in treating breast and pancreatic cancers that resist drug treatment, leading to increased Tumor penetration and low systemic toxicity (59, 60).

**Figure 1:**
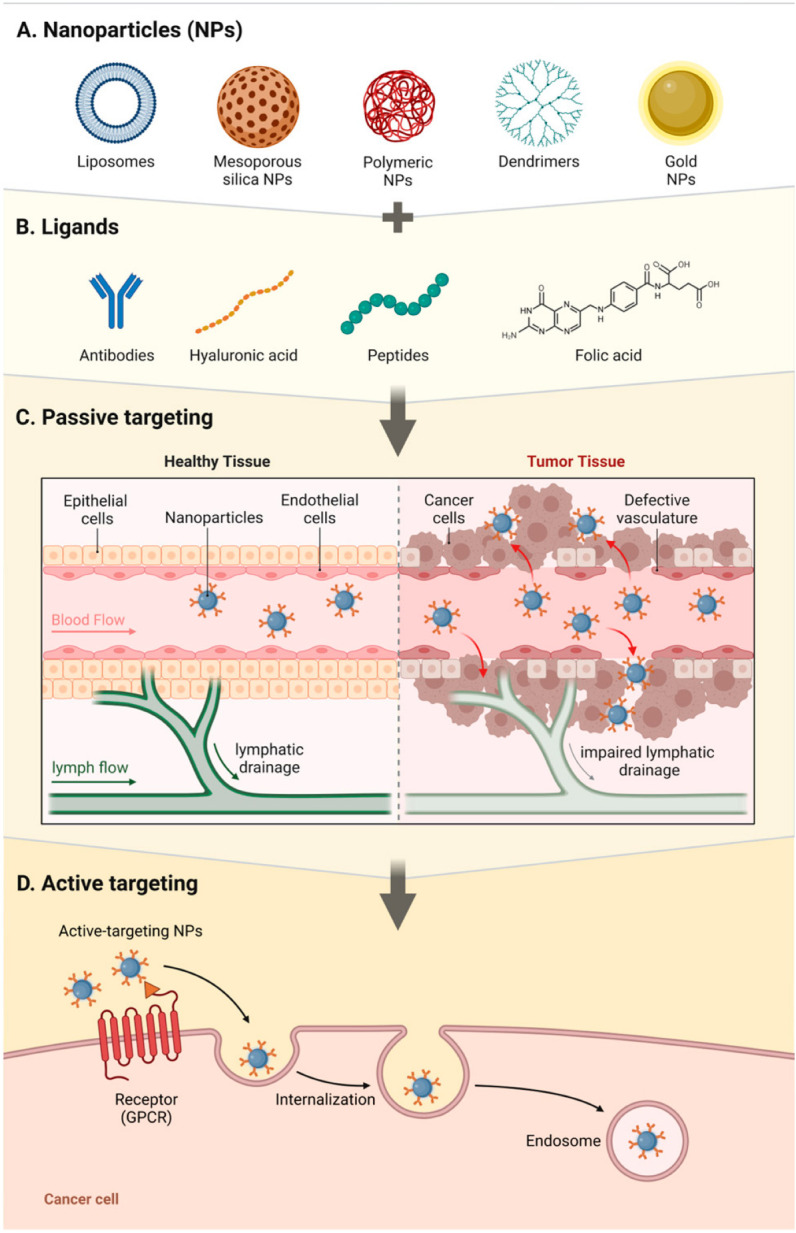
Illustration of several kinds of nanoparticles used for active and passive targeting of tumors with the help of the EPR effect (52). **(A)** Types of nanoparticles used as drug carriers. **(B)** Common ligands used for surface functionalization. **(C)** Passive targeting via the enhanced permeability and retention (EPR) effect in tumor tissue. **(D)** Active targeting through lugand-receptor-mediated internalization into cancer cells.

Active targeting attempts to maximize the therapeutic index by delivering drugs efficiently to Tumor sites while limiting distribution to healthy tissue sites (61). Although there are still problems, such as heterogeneous targets and ligand immunogenicity, active targeting remains superior to passive targeting methods. The development of novel ligands and multifunctional nanocarriers makes active targeting increasingly attractive, facilitating the design of better personalized treatments for cancer patients with more accurate TME targeting (62). **Figure 1** shows different types of nanoparticles used for active and passive tumor targeting based on the EPR effect.

### 2.3 pH-responsive nanocarriers

Tumor microenvironment depends on the presence of acidity in the range from 6.5 to 6.8 because of the Warburg effect, when cancer cells prefer to undergo anaerobic respiration regardless of the oxygen presence. It results in differences between the tumor microenvironment (pH = 6.5-6.8) and normal tissue microenvironment (pH = 7.4) (63). Although pH-responsive nanoparticles remain stable and intact during circulation, their physicochemical properties are altered upon exposure to the Tumor Microenvironment with acidosis, leading to the accurate delivery of the payload (64).

**Figure 2:**
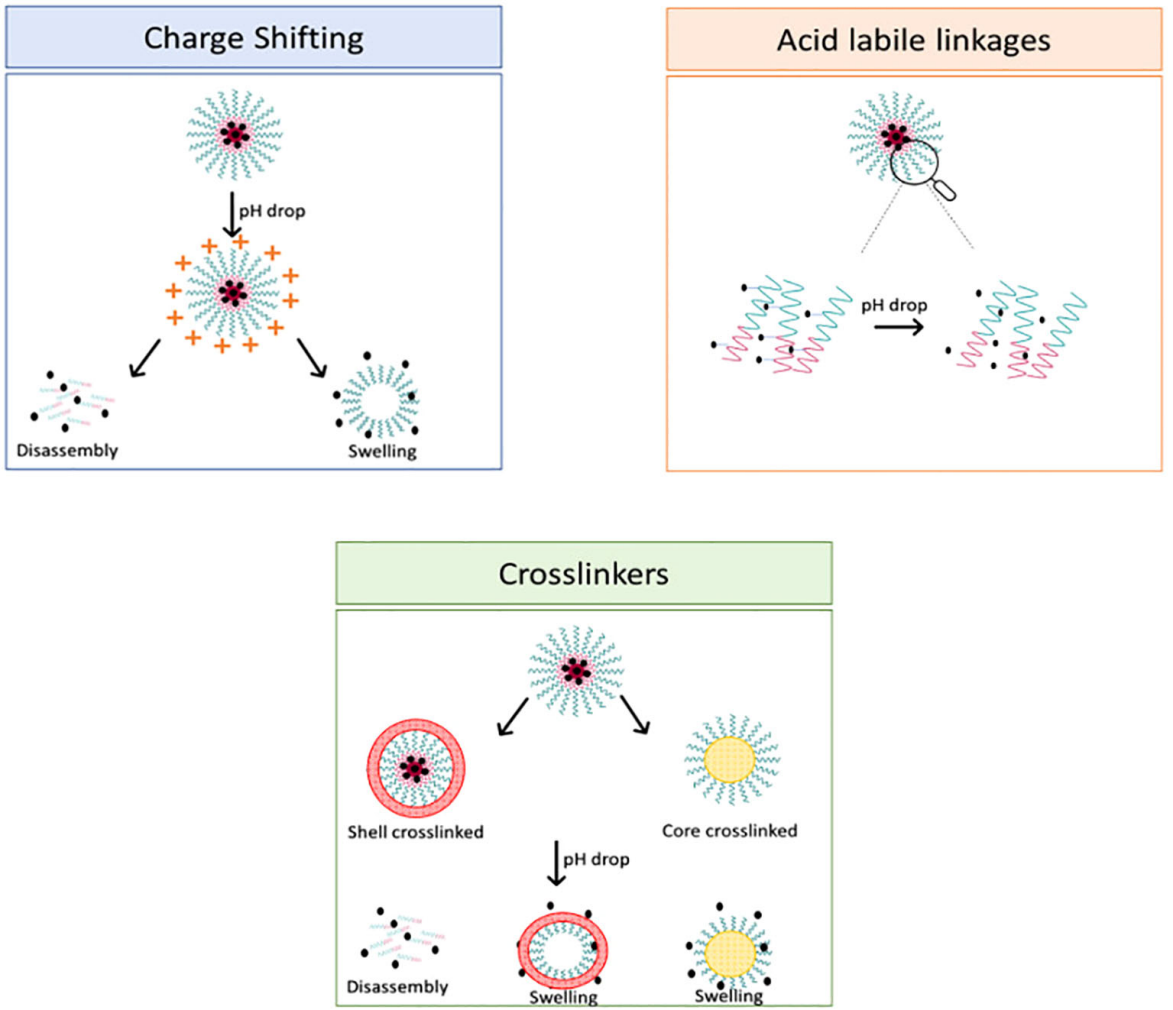
Techniques of producing enzyme-responsive NPs (64).

The design of these nanocarriers makes use of pH-sensitive components. For instance, there have been instances where polymers bearing ionizable functional groups like poly (β-amino esters) and chitosan have been used as they get protonated in acidic environments hence swell up or degrade the nanocarriers (65). An example would be the use of an acid labile chemical bond like hydrazone and acetals to conjugate the drug onto the nanocarrier. This bond is highly stable under neutral pH conditions but degrades rapidly under the acidic conditions inside tumors (pH range of 4.5 to 6.0). This has been shown to lead to efficient delivery of the drug in the targeted regions (66, 67). Recently, pH-responsive mesoporous silica nanoparticles have been shown to have high therapeutic efficacy than non-responsive counterparts when they are loaded with drugs since they can inhibit premature drug release due to the presence of gatekeepers (68–70). **Figure 2** illustrates the strategies used to design pH-responsive nanoparticles.

### 2.4 Enzyme-responsive nanocarriers

The tumor microenvironment is characterized by the abnormal production and activity of several enzymes that are essential for tumor growth, progression, and metastasis. The altered enzyme system represents an accurate signaling pathway for triggering the release of drugs into the Tumor microenvironment (71). MMPs, cathepsins, and hyaluronidase are commonly overproduced in the extracellular matrix or on the membrane of cancer cells, while their level in normal tissues are usually low (72). In the design of enzyme-sensitive nanoparticles, this feature can be strategically exploited. Such agents remain stable and inactive during circulation in the bloodstream, thereby reducing the likelihood of premature drug release and associated toxicity. However, upon reaching the tumor microenvironment, they respond to the presence of specific enzymes, enabling the targeted release of their therapeutic payload at the site of tumor development (73).

By reducing off-target effects and increasing local drug concentration, this technology enhances the treatment index (74). The fundamental approach for generating enzyme-responsive nanocarriers includes incorporating enzyme-cleavable substrates in the structure of nanocarriers. Usually, these substrates include peptides or ester links, which can specifically cleave and hydrolyze under the action of enzymes related to Tumors (73). A protecting polymer layer such as polyethylene glycol (PEG) can be coated onto nanocarriers with an MMP sensitive peptide connection. In blood circulation, the PEG layer prevents the nanocarriers from opsonization and increases their residence time in the body. Upon arrival at the Tumor site, the higher amount of MMP enzymes in the vicinity can hydrolyze the peptide linker, leading to the exposure of nanocarrier or target ligand (75, 76).

Another method involves direct conjugation of the drug to the nanoparticle using an enzyme-sensitive linker (77). Sophisticated nanostructures responsive to enzymes have been created in order to achieve multifunctionality in several stages. One example involves gated nanocarriers, where pores in nanoparticles such as mesoporous silica are covered with polymer or protein material, which could be degraded by enzymes (78).

**Figure 3:**
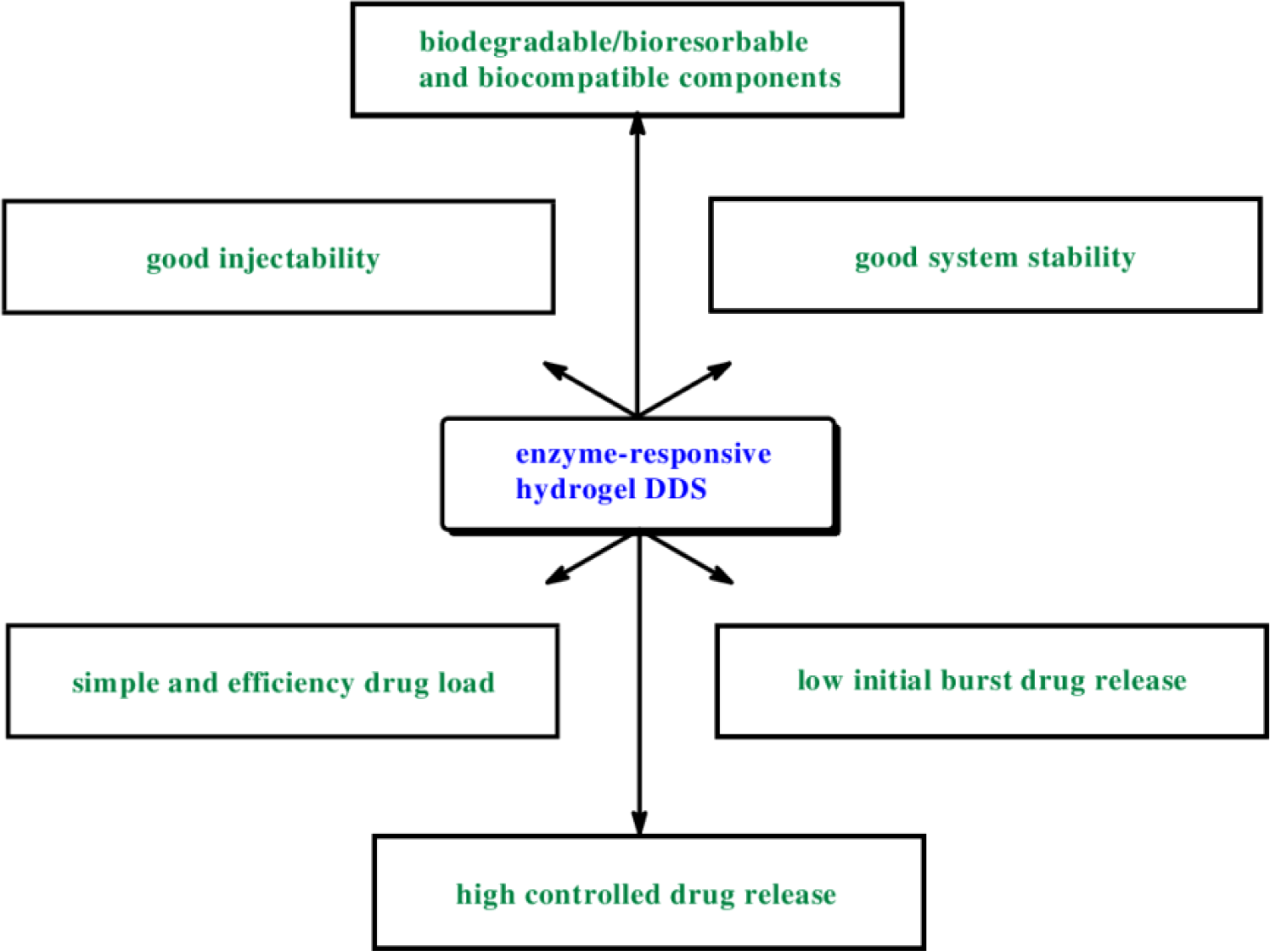
An optimal enzyme-responsive hydrogel drug delivery device (79).

**Figure 3** shows the structure of an improved nanocarrier based on an enzyme-responsive hydrogel for controlled drug delivery. The overexpressed enzymes in the Tumor microenvironment act as catalysts that degrade the covering materials and release drug substances. Such an approach provides highly effective control over the release process. Furthermore, the enzyme responsiveness has become relevant to diagnostic applications as well (80). Thus, allowing the visualization of enzyme activity in Tumor microenvironment along with its location, theranostic nanocarriers could provide imaging signals due to enzymatic cleavage and facilitate the planning of the therapy as well as the analysis of the results (81). Despite some problems related to enzyme heterogeneity within and between Tumors, a high specificity of enzyme-substrate interactions makes the approach an attractive one to study (82).

### 2.5 Hypoxia-responsive nanocarriers

Hypoxia is a key feature of the tumor microenvironment, characterized by a significant deficiency of oxygen resulting from the rapid proliferation of cancer cells outpacing the formation of new blood vessels (83). Hypoxia is strongly correlated with the aggressiveness of cancer, metastasis, and pronounced resistance to chemotherapy and especially radiation therapy requiring the presence of oxygen for its cytotoxicity (84). This unique biological feature can become an internal signal for applying nanocarriers for targeted therapy. Hypoxia-sensitive nanocarriers specifically respond to the hypoxia of the tumor microenvironment and deliver drugs into the most therapy-resistant population of cells. The working principle of hypoxia-sensitive nanocarriers is based on the incorporation of special chemical structures that react only under hypoxic conditions. For example, it is common practice to use nitroimidazole or any other nitroaromatic groups that are stable in normal tissues and sensitive to reduction under hypoxic conditions by nitroreductases highly expressed in hypoxic cells. Bio-reductive reaction may result in the release of a linker carrying the drug and the change in the carrier’s hydrophilicity causing swelling and dissociation of the drug cargo (85).

The existing approach involves azobenzene-containing polymers; upon exposure to hypoxia, the hydrophobic azobenzene moiety is switched to its hydrophilic counterpart—aniline derivative, leading to structural disintegration and cargo release (86). Hypoxia-sensitive drug carriers offer enormous clinical promise, as they target important problems associated with cancer treatment. Using these carriers to deliver cytotoxic drugs and/or HAPs to areas with low oxygen content helps eliminate those cancer cells which cannot be destroyed via traditional means.

**Figure 4:**
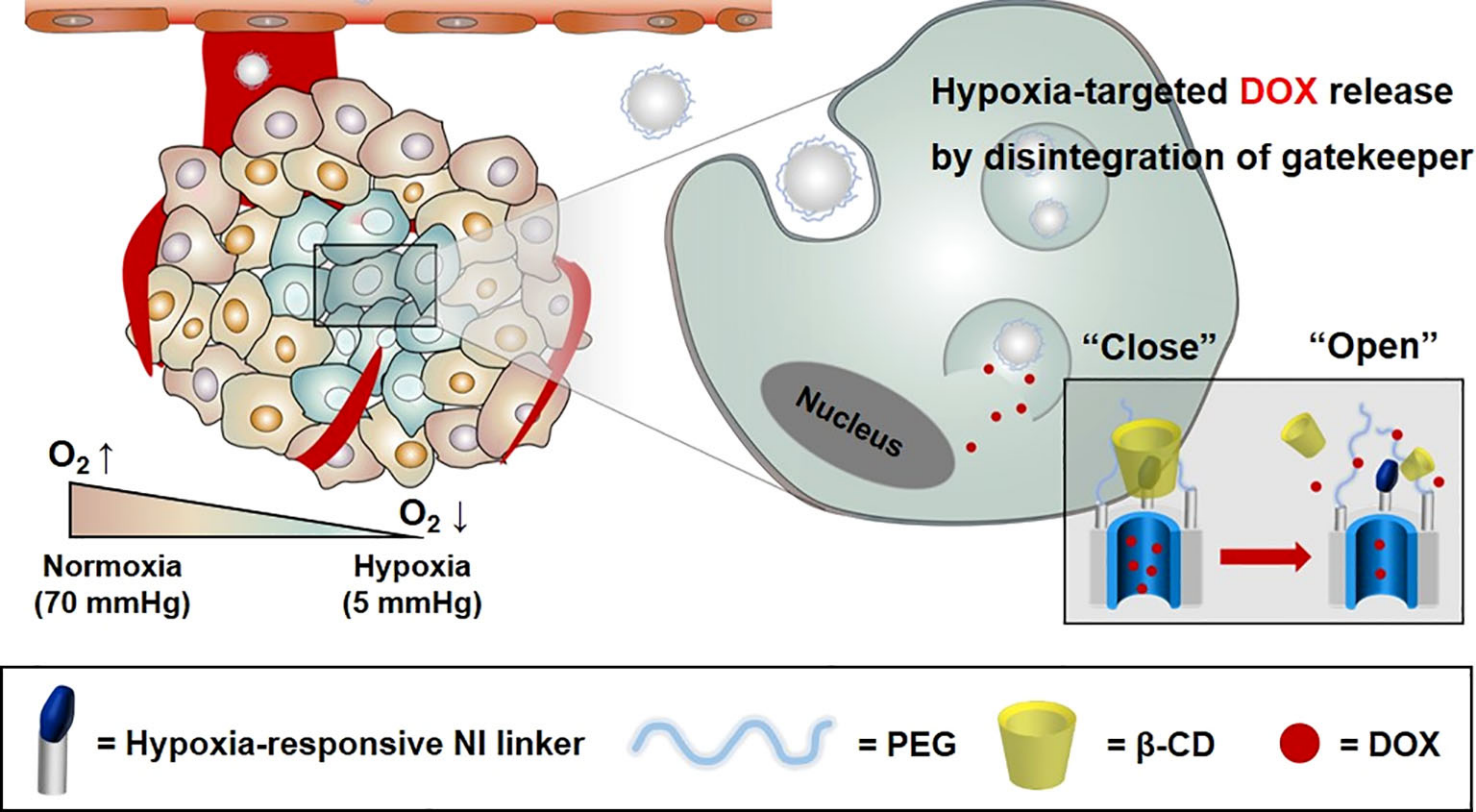
Schematic representation of doxorubicin-loaded hypoxia-responsive mesoporous silica nanoparticles with a β-cyclodextrin gatekeeper (DOX-CD-HR-MSNs) (91).

Furthermore, these carriers may be used to deliver radiosensitizers in order to make radiotherapy more efficient in treating resistant tumors (87). Preclinical studies have shown that hypoxia-responding delivery systems increase the ability of drugs to penetrate deeply into tumor tissue and cooperate with other treatment modalities (88–90), which may lead to a better combination therapy of solid tumors. **Figure 4** shows an example of a hypoxia-sensitive mesoporous silica nanoparticles carrying doxorubicin and gated by β-cyclodextrin (DOX-CD-HR-MSNs). This example represents the use of stimulus-sensitive carrier, which releases DOX under hypoxia conditions within a tumor.

## 3 Mechanisms enabling universal activation of stimuli-responsive nanocarriers despite tumor heterogeneity

Tumor heterogeneity creates one of the most challenging barriers in nanomedicine because stimuli such as acidic pH, hypoxia, and overexpressed enzymes vary not only between tumor types but even between regions of the same tumor. Despite this complexity, stimuli−responsive nanocarriers continue to show therapeutic benefit across diverse patient populations. This is feasible due to the fact that the functioning of such systems does not depend on the exact value of the stimulus; instead, they make use of consistent physiological discrepancies common to cancer tissues. To put it simply, even though the intensity of each stimulus differs, the existence of at least some level of pathological deviation is almost ubiquitous in solid malignancies (92, 93).

In a similar fashion, hypoxia-responsive nanocarriers take advantage of hypoxia and not necessarily the precise level of oxygen concentration inside the tumor. While levels of hypoxia differ immensely from one sample to another, almost all solid tumors have areas where there is not enough perfusion, resulting in hypoxic or quasi-hypoxic areas (94). Therefore, it is possible to utilize nanocarriers that respond to decreased partial pressures of oxygen successfully in most patients.

The case is also applicable to enzyme-responsive nanocarriers. There are enzymes such as MMP-2, MMP-9, cathepsin B, and hyaluronidase, which are over-expressed in almost all metastatic and invasive cancers; however, while their concentrations vary greatly from one cancer type to another, their level is still higher in cancer cells compared to that in healthy tissues (73). In addition, most of the stimuli-responsive delivery systems rely on multiple or overlapping triggering pathways. As a result, they are relatively independent of any particular stimulus. For example, a nanocarrier could employ both pH and enzyme-based triggering methods or be activated by pH and oxidation-reduction potential. Even if one pathway is inadequate or insufficient in a certain tumor, the second pathway could compensate for it (95).

A further design aspect that enables a carrier’s wide applicability potential is threshold-free activation kinetics. Rather than depending on a clear ON/OFF switch occurring at an exact stimulus level, many stimuli-responsive carriers display gradual activation behavior. In the case of pH-sensitive polymers, for instance, a drop in pH gradually destabilizes the carrier instead of demanding a certain level of pH value. Such a mechanism mitigates the influence of differences between patients and increases the carrier’s functional versatility. The physicochemical stability of nanocarriers’ materials is also part of their universal function across various patients due to the wide ranges within which polymers, lipids, and inorganic nanocarriers are made responsive, despite changes in TME (74, 79).

Moreover, most stimuli-responsive carriers are biologically amplified such that, through features like protein adsorption and ion gradients, they are stimulated beyond the minimum requirements needed for activation even with the presence of TME stimulus at relatively low levels.

Several responsive delivery systems also capitalize on the inherent spatial heterogeneity of the tumor. As the penetration of deep tissue areas can be restricted, nanocarriers can activate in response to mild acidity or hypoxia near the edges and later diffuse or release their content to the deeper layers. In this way, delivery to the tumor core is ensured despite the heterogeneous nature of the tumor stimulus profile (96).

Even though the EPR effect is highly heterogeneous, it still acts as a baseline factor leading to a preferential accumulation of many types of nanocarriers. The presence of enhanced local concentration even in cases where the EPR effect is weak will increase the chances of stimulation and consequently trigger the activation of the responsive delivery system (97).

Finally, self-enhancement is common among many stimuli-responsive nanocarriers. Once part of the nanocarrier population activates in response to stimuli, the local environment changes due to the release of the drug, enzymatic cleavage, or other effects. These changes create new stimuli, allowing neighboring particles to undergo similar changes (98).

Significantly, most stimuli-responsive systems are targeted at characteristics that are unique to the diseased tissue but not necessarily the level of disease observed in the tumors themselves. This is not an exercise in accuracy; it is merely enough for there to be a difference between the tumor environment and healthy tissue that allows the drug-delivery system to recognize its abnormality and respond. Another key concept in stimuli-responsive designs is the reliance on universal hallmarks of tumors for their activation. In this regard, the acidic environment, abundance of glutathione, high levels of reactive oxygen species, and enzymatic activity represent fundamental aspects of cancer biology. Although these characteristics might differ in quantity, they exist universally in terms of presence.

Additionally, it is quite common for nanocarriers to include tunable or semi-tunable elements in the design of such systems. For instance, hydrophobicity of polymers, cross-linking density, or enzyme-cleavable peptide size can be regulated to ensure responsiveness throughout the entire pathological spectrum without any need for patient-specific modifications. Such an approach guarantees universality of the resulting product (92).

Many nanocarriers possess buffering abilities, ensuring that they will still respond despite changing environment. This is especially relevant in case of tumors because of pH or oxygen variations due to continuous growth, necrosis, or treatments (70).

It should be noted, however, that it is intensity which varies the most between patients. Well-designed stimuli-responsive nanocarriers make use of the abnormal environment. As a result, regardless of how much of a deviation there is, responsiveness can still be achieved without customization of the system. Moreover, clinical translation approaches often involve combining such systems with diagnostic tests, which help to determine whether or not the target stimulus exists within the tumor prior to therapy.

The most recent developments in the field of nanocarriers have included adaptivity – in that the nanocarrier partly reacts to stimuli in its environment but is completely activated only upon internalization or exposure to intracellular stimuli. The multi-layered nature of responsiveness helps in making sure that the nanocarrier functions properly in cases where the external environment is not conducive (99).

This means that the stimuli responsive nanocarriers will work on different patients without the need for reverse engineering since they have been developed to react to wide pathological parameters, use overlapping stimuli, have gradual activation kinetics, take advantage of universally identifiable hallmarks of cancer cells, and respond to both extracellular and intracellular stimuli. Since they depend on universally identifiable deviations in the TME, there is no need for any specific patient-based redesigning (94).

## 4 Overcoming tumor microenvironment-associated barriers with nanocarriers

### 4.1 Nanocarriers for overcoming extracellular matrix barriers

An important physical barrier that might interfere with successful nanomedicine delivery to solid tumors includes the ECM. Tumor ECM is more rigid than the ECM in normal tissues since it contains an excess of components such as collagen and hyaluronan (100). This rigidness not only serves as a physical barrier hindering the movement of nanoparticles but also increases the IFP that interferes with the convective delivery of drugs from blood vessels to the tumor tissue (101). As a result, drugs accumulate near the tumor vascular system while cancer cells located in deep regions of the tumor tissue, especially the central ones, fail to receive sufficient medication leading to tumor recurrence. A number of strategies have been proposed for nanocarrier design aimed at promoting their permeation through the ECM. An important strategy involves improving the physiochemical properties of the nanoparticles especially their size and charge. Nanoparticles with smaller sizes (<50 nm) can penetrate well in ECM due to their small pore size.

To resolve this problem, several approaches have been proposed for nanocarrier design to facilitate ECM permeation. The improvement of physicochemical properties of the nanoparticles, especially their size and charge, has been suggested to be one of these approaches. Smaller nanoparticles (less than 50 nm in size) show increased diffusion capacity in the dense ECM matrix pores in comparison with larger ones (102, 103). In addition, modification of the nanocarrier surface for creating a neutral or negative charge, usually by PEGylation, allows decreasing the electrostatic and non-specific interactions between nanoparticles and ECM, thus ensuring faster interstitial movement. “Active” nanocarriers that are able to remodel or react to the ECM environment represent advanced techniques (104).

**Figure 5:**
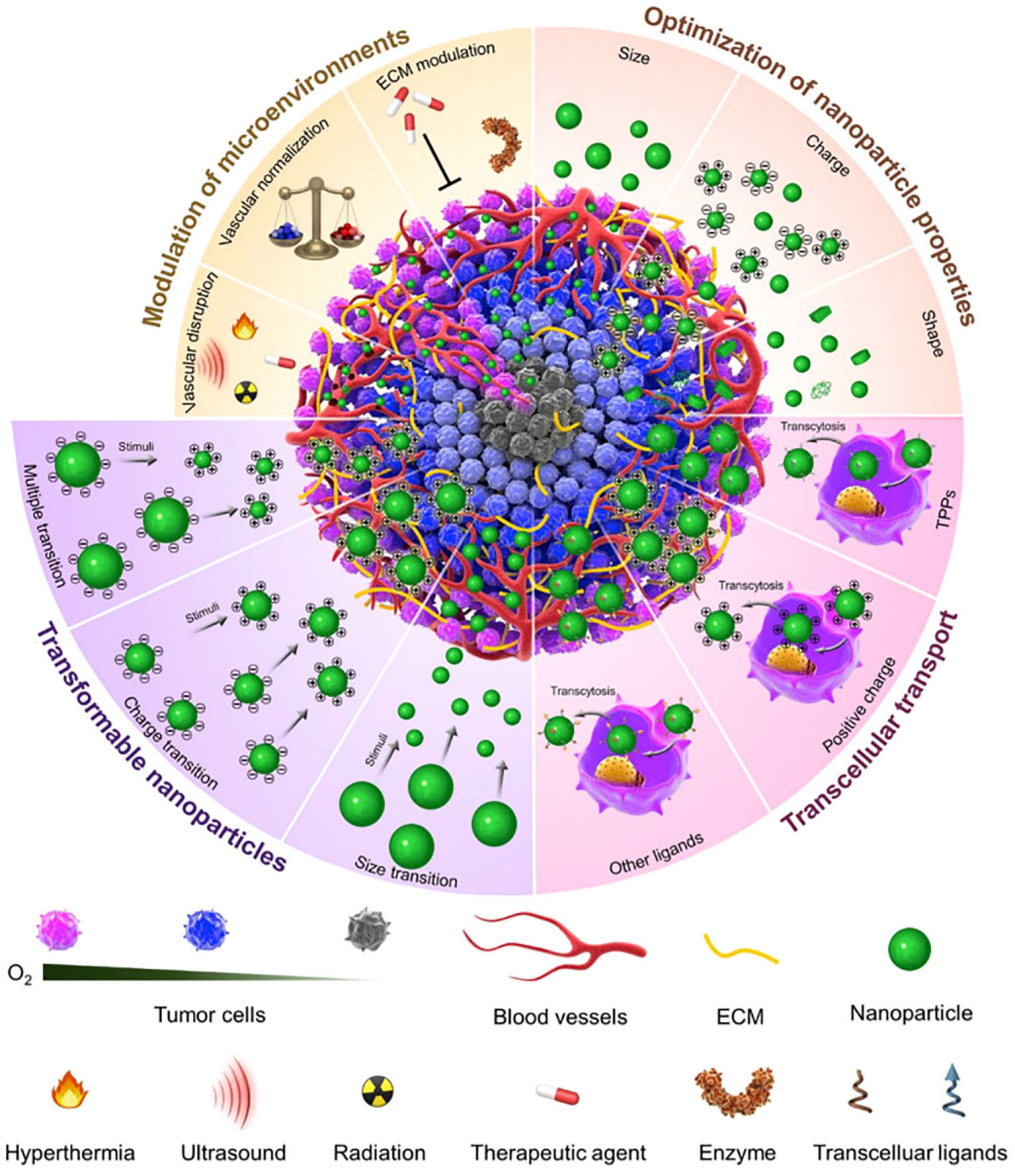
Strategies aimed at improving the distribution of nanomedicine in tumors, mainly via the design of nanoparticles and the manipulation of the TME. Reproduced with permission from (18).

One approach would be to functionalize nanocarriers with enzymes capable of breaking down components of ECM such as collagenase and hyaluronidase. Following arrival at the tumor margins, these nanocarriers release enzymes causing degradation of the matrix, thus creating pathways for penetration into the tumor bed for themselves or any co-administered therapeutic agents (104–106). Another advanced method would include smart systems that alter their properties once inside the TME. This includes nanoparticles that have been designed to shed their coating in response to TME-specific enzymes such as MMPs. Large sizes facilitate circulation and uptake through EPR effect, followed by shedding of coating and reduced particle size allowing easier diffusion into tumor tissue (104–106). Strategies for enhancing infiltration of nanomedicine via adjusting nanoparticle and TME properties are shown in **Figure 5**.

### 4.2 Nanocarriers to overcome immune suppression

The microenvironment of a tumor is commonly marked by a highly immunosuppressive state, which poses a major challenge in terms of anti-cancer immunity and a significant cause of failure in immunotherapy treatments (107). An increase in the population of T regulatory cells (Tregs) and M2-polarized TAMs, an elevation in the expression levels of immune checkpoints such as PD-L1 on tumor cells, and the secretion of immunosuppressive cytokines such as TGF-β are some factors responsible for the generation of this immunologically hostile microenvironment (108, 109).

This combination of factors serves to inhibit the activity of effector cells, particularly cytotoxic T lymphocytes (CTLs), thus restricting their capacity to recognize and destroy cancer cells. Nanotechnology provides an efficient approach for dynamically manipulating the immunosuppressive microenvironment and shifting the balance from immunological tolerance to robust anti-tumor immunity (110). Nanovehicles can be engineered to manipulate the tumor microenvironment by administering immunomodulatory agents with high precision and reduced systemic toxicity.

**Figure 6:**
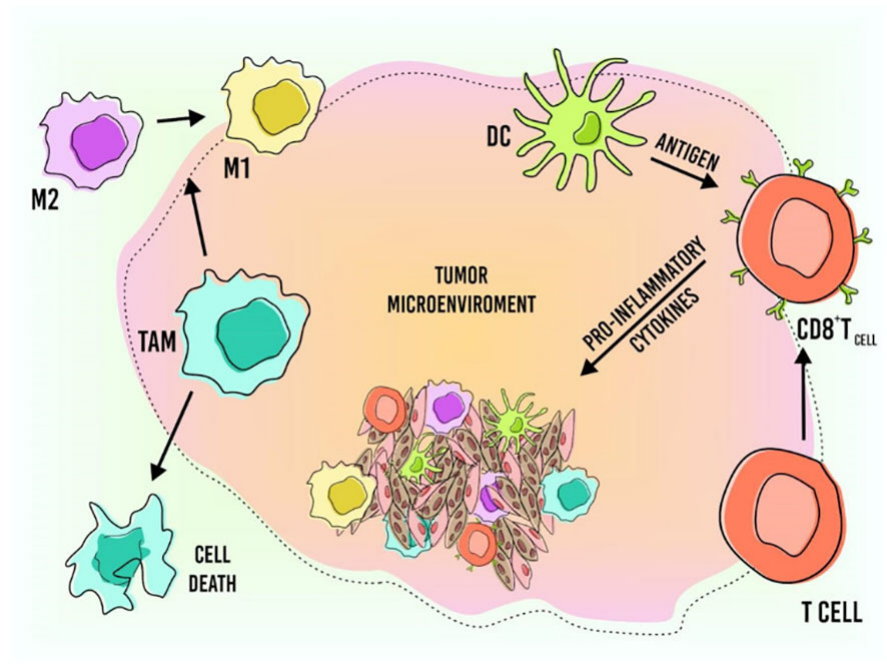
Immune cells linked to the tumor microenvironment and potential consequences after contact with nanoparticles (112).

One of the key strategies is the controlled use of immune checkpoint inhibitors (ICIs), which involve targeting PD-1 or PD-L1 receptors through antibodies. The encapsulation or attachment of these drugs to nanoparticles will improve their delivery to the TME, increasing their effectiveness within the tumor tissue and minimizing immune-related adverse effects on healthy tissues (53, 111). An illustration of different immune cells found within the TME and their interaction with nanoparticles is shown in **Figure 6**. Nanoparticles can be designed to selectively target and manipulate immunosuppressive cells. For instance, nanoparticles loaded with drugs capable of converting pro-tumor M2 TAMs to anti-tumor M1 TAMs may considerably change the immune composition within the TME. Similarly, nanoparticles containing siRNA can be used to block genes responsible for producing immunosuppressive substances, such as TGF-β and STAT3, interfering with crucial mechanisms involved in evading the immune system (113, 114).

The use of nanomedicine through its ability to elicit ICD and increase immune cells’ infiltration can help reverse suppression of the local immune cells and induce a strong and global anti-tumor immune response. The cancer cells can generate DAMPs and TAAs due to immunogenic cell death (ICD) caused by delivery of chemotherapy drugs through the use of nanocarriers, e.g., doxorubicin and oxaliplatin (115, 116). This process recruits and activates antigen presenting cells, like DCs, thus creating an *in situ* vaccine. An adjuvant, for example, TLR agonist, which is co-delivered along with the chemotherapeutic drug by the nanocarrier, further intensifies the effect (117).

This mechanism facilitates maturation of DCs and consequent activation of tumor-specific cytotoxic T cells that infiltrate and kill tumor cells (118). Through these mechanisms, immunologically “cold” and unresponsive cancer becomes “hot” and sensitive to immune system attack through nanomedicine (119).

### 4.3 Reducing interstitial fluid pressure with nanocarriers

**Figure 7:**
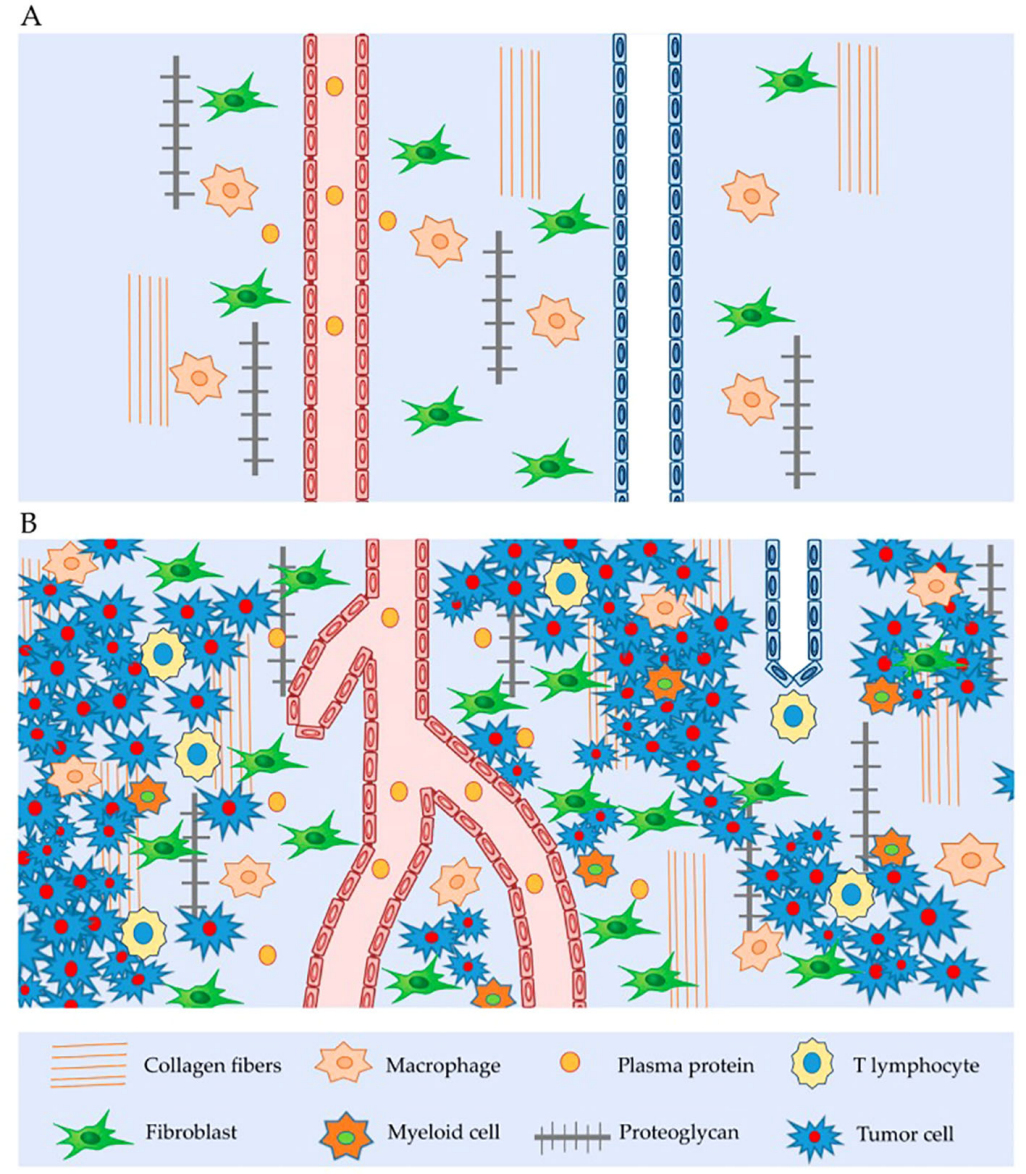
Interstitium tissue: **(A)** Normal interstitial tissue with lymphatic and blood vessels, macrophages, fibroblasts, collagen fibers, and proteoglycans. **(B)** Tumor interstitial tissue characterized by diminished lymphatic vessels, convoluted blood vessels, macrophages, fibroblasts, an elevated presence of extravasated plasma proteins, T-lymphocytes, myeloid cells, collagen fibers, proteoglycans, and tumor cells, all contributing to an increase in tumor interstitial pressure (121).

One of the most significant physiological barriers in solid tumors is the elevation of interstitial fluid pressure (IFP), which significantly affects the transport of drug molecules (120). The elevated interstitial fluid pressure, which can reach the levels of arterial blood, is caused by the combination of the permeability of the tumor blood vessels and the inadequate drainage of the lymph fluid from the tumor. The formation of an IFP gradient causes the generation of a strong outflow of fluids from the center of the tumor towards the peripheral areas of the tumor, and thus, inhibits the influx of medicines through nanocarriers to the tumor tissue (18). Consequently, the accumulation of drugs in the tumor area is not sufficient for the successful treatment process (18). **Figure 7** shows the structure of normal and tumor interstitial tissues.

The use of nanocarrier-based approaches has been developed to modulate the tumor microenvironment and facilitate better delivery of drugs used in treatment. One of the simplest approaches that can be employed in an effort to alter physical dynamics in the tumor microenvironment is through “vascular normalization” with the aid of nanocarriers. As proposed in this approach, nanocarriers deliver a low-dose anti-angiogenic drug to “normalize” tumor vasculature by inducing maturation rather than regression of vasculature (122). The delivery of nanocarriers loaded with drugs like anti-angiogenic agents such as VEGF inhibitors and tyrosine kinase inhibitors will help increase the stability of cell junctions and also increase the number of pericytes covering the vessels. This action leads to decreased permeability of the vessels, resulting in reduced fluid extravasation in the interstitium, which in turn lowers IFP (123, 124). This process increases the ability of the drugs loaded in the nanocarriers to infiltrate the tumor and also oxygenation of the tumor cells, leading to increased sensitivity to treatment such as radiation (125, 126).

In addition to their effects on the vasculature, nanocarriers can also reduce IFP by modifying the thick tumor stroma, which induces mechanical compression of intratumoral blood vessels and high solid stress. Nanocarriers with enzyme-loaded cargoes, such as collagenase and hyaluronidase, may reduce the associated mechanical stress as discussed before (Section 3.1) (106). These drug-delivery systems aid in degrading the ECM, creating “decompressed tumor”. This could release mechanical stress in vessels, including blood and lymphatic vessels, resulting in a decrease in the IFP and fluid flow (127).

Moreover, there are efforts towards the development of advanced nanocarrier technologies targeting CAFs, which play a critical role in stromal deposition and VEGF synthesis. The treatment using inhibitors of cancer-associated fibroblasts (CAFs) via nanocarriers is a strategy that could simultaneously reduce ECM density and proangiogenic signaling (128–130). As a result, the normalization of both physical and angiogenic components of the TME will be achieved.

## 5 Multifunctional nanocarriers for chemotherapy and beyond

### 5.1 Co-delivery of chemotherapeutics and gene therapy

Despite many innovations and discoveries made in the twenty-first century, cancer still remains one of the most significant causes of mortality around the globe. Chemotherapy treatment is widely used in various kinds of malignant tumors; however, due to its multiple applications, it may become toxic, non-specific, and develop multidrug resistance (MDR), thus reducing its effectiveness (131, 132). The solution to the stated problem is represented by combination therapy treatment options which have been well-researched and applied in cancer cases effectively. Cancer results from several mutations, while its development is characterized by a number of genome alterations and disorganized signaling pathways (133).

Mutations affect cell-cycle advancement, apoptosis, and cell proliferation; thus, they play an essential role in the formation and development of cancer (134). The combination therapy approaches involving the use of CRISPR/Cas9 genome editing or siRNA treatment along with chemotherapy appear to be highly beneficial in this case (135).

This delivery method tries to act on multiple pathways involved in the tumor formation and resistance mechanism at once, hoping to achieve synergic effects. It is not easy to look for the best carriers for drug and gene co-delivery because, on the one hand, most chemotherapy drugs are hydrophobic small-molecule substances, while gene drugs tend to have a higher molecular weight and negative charge (136).However, the co-delivery system has made tremendous progress over the past few years.

These two components cannot be encapsulated using the same process due to their significant differences in physicochemical properties; the former usually relies on the compression of carriers with an electrostatic force, while the latter may make use of an electrostatic force, hydrophobic force, or chemical conjugation inside nanocarriers (137). To resolve this problem, various traditional nanocarriers, like liposomes and micelles, as well as novel nanocarriers, like dendrimers and supramolecules, have been applied for the delivery of chemotherapeutic drugs and gene agents (136, 138).

Many researchers have examined various nanocarriers for the concomitant delivery of both chemotherapeutic and gene-based therapeutics, each exhibiting distinct properties and advantages. Recently, intelligent nanoparticles that co-delivered chemotherapeutic drugs such as fluorouracil and oxoplatin (FuOXP) along with siRNA against Xkr8, an anti-apoptotic gene responsible for protecting tumors from the immune system, were investigated. It was discovered that intravenous injection of the nanoparticle in colon and pancreatic cancer models effectively inhibited tumor development and induced an anti-tumor immune response (139).

Another interesting property of the highly branched DNA nanostructure is its ability to be used in designing drug carriers. An innovative approach of DNA dendrimers as an intelligent delivery system was demonstrated in 2025. In this work, DNA double helices are employed as platforms for loading antisense oligonucleotides along with water-soluble and insoluble small molecule drugs. For co-delivering the therapeutic agents with regulation through targeted drug release, this work proves that drug nanocarriers loaded onto DNA dendrimer structures are capable of reaching cancer cells via endocytosis and releasing drugs in response to glutathione in intracellular environments. This provides a promising method for drug delivery in Tumors (140).

Li et al. carried out another experiment where they formulated FTD NPs consisting of trimethyl chitosan that were folic acid-and DPA-functionalized for the co-delivery of doxorubicin and plasmid encoding CRISPR/Cas9 against Survivin. These improved nanoparticles appeared to be more effective in their action against tumor compared with DOX and sgSurvivin treatments individually, making it a potential solution for treating cancer (141). Furthermore, an oncology-targeted nanosystem based on DOX-loaded core and PAMAM–RGD decorated with siRNA against c-myc has been designed to address glioblastoma cancer. This nanosystem was shown to have the potential to provide a synergetic effect in the treatment of cancer owing to increased uptake, cell selectivity, and ability to cross the blood-brain barrier (142).

### 5.2 Nanocarriers for immunotherapy and chemotherapy combination

Unfortunately, at present, not many effective methods for the treatment of cancer exist. Nowadays, immunotherapy, which focuses on eliminating tumor cells using the immune system of the patient, is viewed as the fifth cancer therapy method following surgery, chemotherapy, radiotherapy, and molecular targeted therapies (143, 144). One clinical study indicated that compared to standard chemotherapeutic agents, CAR-T cell immunotherapy proved to be a superior therapy strategy for various hematological malignancies (145). Despite the high prospects of immunotherapy in cancer treatment, its efficiency as an independent cancer therapy remains relatively poor (146). To resolve this issue, the use of chemoimmunotherapy (CIT) as a combination of classic chemotherapy with novel immunotherapies can be implemented to halt tumor growth, metastasis, and relapse (147).

In addition, although earlier studies showed that chemotherapy has negative impacts on immune responses, recent findings have demonstrated that the process might actually facilitate immunotherapy. In detail, the ability of the immune system to detect malignant cells increases, tumor antigens and immune checkpoints become more apparent, tumor microenvironment changes, and some immune blockade effects are reduced (148). Unfortunately, the efficiency of CIT in treating solid tumors remains low despite its outstanding effectiveness in hematological malignancies (149, 150).

Even though CIT is an attractive strategy for tumor therapy due to its reduced risk of recurrence, the utilization of this approach may be restricted due to short half-life, low accumulation, limited penetration into the tumor tissue, and cytotoxicity in physiological environments. The development of nanotechnology could help solve these problems, reducing both the dose and the number of administration, thereby providing a completely new way for CIT to become safe. In recent years, multifunctional nanomaterial-based drug delivery systems (NDDSs) have been increasingly used; their flexibility and versatility give them great potential in dealing with various types of cancer (146).

Pham et al. synthesized PD-L1-targeting paclitaxel (PTX)-loaded albumin nanoparticles (PD-L1/PTX@HSA) via the use of nanoparticle albumin-bound technology. It was demonstrated that the synthesized nanoparticles effectively inhibited tumor growth both *in vitro* and *in vivo* by modulating the regulatory T-cell population, enhancing effector T-cell recruitment, and influencing the immunosuppressive protein Programmed death-ligand 1 (PD-L1). They can be considered an anti-tumor agent, having no toxic effects on organs (151).

Another study involved the delivery of doxorubicin, two immune adjuvants, and one chemokine (CCL20) with biodegradable nanoparticles as treatment for refractory solid tumors. Solvent evaporation-extraction was used for nanoparticle preparation with immune adjuvant loading along with doxorubicin and NIR dye. These nanoparticles proved to be highly effective against two models of treatment-resistant cancers (TC-1 lung carcinoma and MC-38 colon adenocarcinoma) with high T cell activation and infiltrating leukocyte population increases. This approach could lead to improved solid tumor treatment (152).

In animal studies, Kuai et al. illustrated that doxorubicin-loaded nanodiscs, which mimic high-density lipoprotein, could enhance the effects of immune checkpoint blockade therapy. The nanodiscs elicit strong CD8^+^ T cell response, induce immunogenic cell death, and improve antitumor efficacy. Nanodiscs induce complete tumor regression and help to prevent tumor relapse while used with anti-PD-1 therapy (153).

A current study incorporated ICD induced by chemotherapy with immunotherapy to improve breast cancer treatment through developing a novel nanoparticle, which delivers paclitaxel, siCD47, and immune adjuvant R848. Such nanoparticles appeared very promising for synergistic chemo-immunotherapy due to their high efficiency in targeting tumors, immune activation, reducing tumor growth, and immune cell population increase (154).

### 5.3 Considerations related to synchronization and synergy in nano-mediated co-delivery of chemotherapy and immunotherapy

Co-delivery of chemotherapy and immunotherapy through nanocarriers appears promising due to the synchronization in tumor cell destruction and immune activation, although recent studies reveal that therapeutic effects are critically dependent on sequence and synergy between these two therapies. The timing of chemotherapy and immunotherapy does play an important role because the interaction between them affects immune activation, suppression, or priming. In the case of stimuli-responsive nanocarriers, the situation becomes more complicated as release from the carrier depends on multiple environmental factors within the TME, including pH, enzyme concentration, and hypoxia (41, 155).

Chemotherapy can lead to immunogenic cancer cell death with increased antigen presentation, promoting dendritic cell maturation; however, too strong or too early delivery of cytotoxic drugs can kill the immune effectors along with tumor cells. Therefore, the desired treatment sequence is that chemotherapy-induced stress occurs first, followed by immune stimulation. Stimuli-responsive nanocarriers capable of sequential release in response to environmental changes can reproduce the desirable sequence.

From experimental results, it is known that when chemotherapy is delivered shortly before immunotherapy, it increases antigen presentation, creating an environment conducive for better action by checkpoint inhibitors or immune agonists. It applies to nanocarrier systems, where cytotoxic molecules released in acidotic or enzymatic tumor microenvironments can precede the delivery of immunomodulating drugs after some delay. It allows for circumventing immune suppressive effects and increasing the antigen priming process (156).

This benefit can be seen when studying immunogenic drugs such as doxorubicin or oxaliplatin that induce calreticulin expression and DAMP secretion before interfering with immune checkpoints. If two kinds of drugs are delivered from a nanocarrier at the same time, the biological window can be missed, thus decreasing synergistic effects. The literature supports that nanocarriers should first interact with extracellular factors in tumor sites and later deliver immunomodulating molecules inside cells or the immune system (157, 158).

Dosage is another important consideration. Chemotherapy through nanocarriers usually results in more efficient accumulation within the tumor tissue and less systemic exposure than free chemotherapy. It enables lower cytotoxic doses to be administered, ensuring immune cells remain alive while continuing to kill tumors. But if chemotherapeutic load is excessive, toxicity might affect the infiltrating lymphocytes or antigen-presenting cells, reducing the potential for immunotherapy (159).

Chemotherapy-to-immunotherapy agent ratio has been found to affect response efficacy. Preclinical studies demonstrate that when there is a ratio that allows the release of tumor antigens while sparing the exhaustion of T-cells and avoiding excessive apoptosis, the immune system produces better responses. The nanocarrier may manipulate the ratio by altering core-shell design, compartmentalization, and conjugation density, although an inappropriate ratio will cause antagonist reactions (160).

The multi-stage nanocarriers with hierarchical structures exemplify how the proper proportionate release may sustain synergies. For instance, a hydrophobic core may encapsulate the chemotherapeutic agent to achieve rapid release in extracellularly acid environments, whereas the hydrophilic shell with immunomodulators remains intact until exposed to intracellularly oxidative environment. Sequencing of such kind is consistent with a recent trend in favor of sequential release rather than simultaneous exposure (161).

The immune profile of the tumor is also significant in determining sequencing requirements. Cold tumors, which lack immune cells, need the antigens to be exposed through chemotherapy for the immunotherapy to be effective. On the other hand, hot tumors have active immune cells that may actually benefit from having multiple drugs available at the same time (162).

Antagonisms due to cytotoxicity occur when the chemotherapy is provided too harshly and before immunotherapy is administered. Although it mitigates the effects, nanocarrier delivery methods do not eliminate this risk entirely because the sudden release of cytotoxics may affect lymphocytes and dendritic cells in the area where they are highly concentrated due to the high acidity or enzymes in the area (163).

Yet another type of antagonism happens when the immunotherapeutic agent activates immune response pathways prematurely. When the immunotherapy is provided without prior antigen exposure or tumor debulking, the T cells may fail to activate effectively. If nanocarriers only allow for one trigger release methods, the simultaneous response could result in misalignment of therapies (164).

The issue of cross talk among intracellular pathways is yet another hurdle. Some cancer drugs trigger apoptotic pathways that inhibit NF κB signaling or disrupt antigen presentation pathways, making immune adjuvant treatment less efficient. Controlled sequential release within nanocarriers seeks to minimize the overlap of intracellular exposure, although partial compartmentalization may cause interaction issues (165).

Another factor influencing the likelihood of antagonism is the pharmacokinetics of the combination therapy. Immunomodulatory drugs usually need sustained presence to function effectively as blockers or stimulators, while cancer chemotherapy drugs work well through pulse and high concentration delivery. In case the nanocarrier cannot handle both pharmacodynamic features, the joint therapy becomes kinetically similar to either one of them (166).

Nanocarriers that can overcome this challenge include asymmetrical physical properties, including Janus particles and anisotropic vesicles, which create physical separation of compartments within the carrier. This strategy minimizes diffusion interference and enables individual responses to tumor microenvironment signals for each class of drugs. When done right, the architecture creates a synergy from antagonistic overlap (167).

Some advanced strategies have been devised where the immunotherapy release is triggered only after detection of chemotherapy-induced biomarkers, like particular DAMPs signatures and ROS levels. This biological requirement helps the nanocarrier avoid triggering premature immunotherapy release. Although promising, such systems are still quite complicated and prone to sensitivity to the spatial heterogeneity of the TME or inadequate induction of ICD (168).

Spatial heterogeneity in the TME is one source of possible antagonism. The accumulation of nanocarriers in hypoxic or necrotic zones may lead to preferential chemotherapy release in these locations while the immunotherapy requires contact with active immune cells, present mainly around tumor edges. Without proper matching of release kinetics to address spatial discrepancies, spatial antagonism might occur regardless of correct timing (169).

Moreover, the release kinetics affected by TME variability influence synchronization of release processes. In one patient, a low level of enzyme activity might trigger the delayed release of immunotherapy that would extend past the chemotherapy-created therapeutic window. Meanwhile, in another individual, quick enzymatic cleavage might result in over-synchronization of releases from multifunctional carriers (170).

However, despite the difficulties discussed above, many studies point to a higher likelihood of synergy versus antagonism through temporal separation and dose balancing of release. Nanocarriers’ ability to target chemotherapy in the tumor and control immunotherapy bioavailability gives them a mechanism not achievable with systemic administration (171).

Furthermore, there is evidence that the use of nanocarriers in co-delivery decreases systemic immunosuppression by avoiding the overexposure of lymphoid organs to chemotherapeutics, ensuring normal immune system functionality and effectiveness of checkpoint blockade or adjuvancy. By mitigating this major factor that contributes to antagonism in systemic chemoimmunotherapy, nanocarriers further enhance their potential for synergy. In addition, current knowledge from preclinical research calls for staggered and ratio-controlled releases based on biological events in co-delivery using nanocarriers. Although antagonism remains a possibility, it is mainly associated with the simultaneous release of two drugs regardless of biological events required for successful activation (172).

Therefore, synergy versus antagonism for nanocarrier co-delivery can be achieved via an optimal combination of three aspects that include temporal coordination based on the kinetics of ICD-induced cell death, proper dosage to preserve immune responses, and TME-responsive release profiles in accordance with tumor heterogeneity. The current scientific data support sequential/multi-stage rather than concurrent release, and contemporary nanocarrier technology is moving towards a more rational approach to achieve such therapeutic synergy (173, 174).

### 5.4 Overcoming multidrug resistance with nanocarriers

There has been extensive utilization of chemotherapeutic agents in cancer treatment. Nonetheless, the effectiveness of anti-cancer drugs in tackling cancerous cells has been compromised due to the development of multidrug resistance (MDR). This kind of resistance results in metastasis, recurrence of cancer, and death of patients (175). There exist several causes of resistance to cancer chemotherapy. These include increased drug expulsion, detoxification, cancer stem cells, epithelial-to-mesenchymal transition, inhibition of drug activity before its action at the target site, suppression of cell death, increased gene amplification and repair processes in oncogenes, in addition to drug metabolism (176).

MDR can be categorized into two major types; intrinsic resistance where the focus is on cancer cells natural ability to evade therapy and acquired resistance where the focus is on progressive development of resistance of cancer to drugs. Combating resistance of the two categories calls for in-depth knowledge of drug resistance targets and routes (177). Overcoming MDR constitutes one of the most significant challenges in cancer chemotherapy. It becomes necessary therefore, to overcome resistance to various kinds of cancer and make cancer cells sensitive to immunotherapy, chemotherapy, and target drugs (178, 179).

This therapy represents an innovative approach for treating Tumors and overcoming Multidrug resistance because this therapy uses nanoparticles that are capable of carrying two or more different dosage forms or sizes of pharmacologically active drugs, which makes it more efficient while decreasing the side-effects associated with chemotherapy (175).

There are many types of nanocarriers, including liposomes, micelles, dendrimers, and diverse inorganic and organic NPs, all having the ability to modify the biodistribution, activity, and even toxicity of the encapsulated compounds owing to their unique chemical, physical, and biological properties (180, 181). Nanoparticle researchers are especially interested in surface modification of nanocarriers to enhance efficacy of anti-cancer drugs (182). Targeting approaches, both passive and active, are used in order to increase pharmaceutical safety, controlled delivery of drugs, and targeted drug delivery (183).

In cancer cells that are resistant to chemotherapy drugs, these drugs enter the cells via diffusion while being expelled by ABC transporters, limiting their efficacy. Cationic polymers capable of targeting the nuclei are highly effective in transporting anticancer drugs within nanocarriers. Consequently, multifunctional nanoparticles containing both anticancer drugs and MDR reversal agents have been suggested as an approach to combating multidrug resistance in tumors (131, 175, 176).

Current research is directed towards developing nanomaterials aimed at targeting ABC transporters for reversing multidrug resistance in tumors. One such study considers using nanoparticles loaded with doxorubicin (DOX) for reversing MDR in HCC. In particular, HepG2/DOX cells and nude mice were used in the experiment to evaluate the effectiveness of the drug. The results from fluorescence imaging revealed that treatment with DOX-nano increased apoptosis in cells, blocked their migration, and inhibited tumor growth. Further, lower levels of MDR proteins were observed in cells and tumors treated with DOX-nano (184).

Multidrug resistance associated with cancer has been proven to be overcome using a biomimetic approach, which incorporates the use of lemon EVs and HRED nanodrugs. This approach penetrates DOX resistant cancerous cells through the mechanism of caveolin-mediated endocytosis, macropinocytosis, and clathrin-mediated endocytosis to dissipate cellular energy, lowering ATP production and thus significantly diminishing drug efflux. As such, they have been successfully applied as an approach to overcoming ovarian cancer MDR through their anti-proliferation activity against DOX-resistant ovarian cancer (185).

A new palmitic acid analogue of docetaxel (DTX), which is an effective anticancer drug, was developed. In-silico studies showed low binding affinity of DTX-PL to the P-glycoprotein, indicating its poor efficacy as a substrate for efflux mediated by P-gp. Solid lipid nanoparticles (SLNs) of various lipids have been applied for conjugation to form a compound. It had shown reduced IC50 compared to DTX, improved solubility, lowered protein binding in plasma and P-gp efflux ability (186).

A research study conducted revealed that there was enhanced intracellular delivery of the nanodrugs because of the covalent binding of the carbon nanotubes of a particular nanoscale size with the drug doxorubicin (DOX). Also, carbon nanotubes-DOX remain in the cellular environment of drug-resistant tumors for a longer period of time and result in mitochondrial dysfunction, ATP synthesis inhibition, and ultimately deliver therapeutic benefit in resistant tumors. A research study conducted revealed that H69AR lung carcinoma cells, which are drug-resistant to the anticancer drug doxorubicin (DOX), are not resistant to the nanoformulation of anticancer drugs of a particular nanoscale (187).

## 6 Modulating tumor microenvironment to enhance drug delivery

### 6.1 pH-driven nanocarriers for tumor-specific drug release

Acidic nature of tumor microenvironment is one of the major causes due to the Warburg effect, which leads to the generation of lactate and H^+^ ions in tumor cells (188). Utilizing the acidic nature of TME, pH-sensitive nanocarriers may be used for the targeted delivery of medications and subsequent drug release at TME sites. In the bloodstream, pH-sensitive nanocarriers may show optimal stability under physiological pH conditions. Under the acidic environment at TME, these nanocarriers will change chemically and physically because of the reduction in pH values by generating gas, changing surface charge, and breaking down at low pH values. Thereafter, drugs are directly delivered into TMEs, or there will be an effective uptake of nanocarriers into cells (189) (190),.

Due to their promising applications in improving drug uptake and increasing treatment efficacy, while minimizing adverse effects, pH-sensitive nanocarriers are expected to produce better clinical outcomes. Advanced pH-sensitive nanocarriers that are less toxic and more efficient have been reported due to the rapid development in nanotechnology in recent years (191). Two major approaches widely employed for the encapsulation and pH-sensitive delivery of active substances are: (a) core–shell systems with acid-cleavable shells and (b) dendritic support-based systems functionalized with solubilizing groups and cleavable linkers. These strategies are designed to achieve controlled, site-specific drug release in response to acidic pathological environments such as tumors, inflamed tissues, endosomes, or lysosomes (192).

To deliver doxorubicin (DOX) into intracellular space, scientists developed a CaCO_3_-core cross-linked nanoparticle, called CaNP/DOX. Its pH sensitivity, uniform size distribution, and ability to carry drugs effectively were proved. Application in mice with osteosarcoma K7 cells demonstrated improved drug delivery, increased drug potency, half-life period in blood plasma, and anti-tumor effects. In such a way, smart drug delivery system was shown its high competitiveness in medical oncology (193).

The nanosystem, designed to achieve “off/on” function in case of synergistic therapy (photo-dynamic therapy and ferroptosis) and multimodal diagnosis with a tumor-specific acidic pH trigger, can be successfully used in overcoming drug-resistance and precise drug delivery and diagnostics for HCC. SR780 is a novel amphiphilic photosensitizer that is based on a croconaine dye. This photosensitizer has unique pH sensitive NIR-II imaging and photo-dynamic activity and can be deactivated in mildly acidic environment by complexation with Fe^3+^ (SR780@Fe) (194).

The collagen hydrogel nanocomposite material (Col-APG-Cys@HHD) was produced by crosslinking collagen with recombinant albumin nanoparticles (HHD NPs). The degradation of the hydrogel under acidic conditions resulted in the release of HHD NPs. The particles are pH sensitive; therefore, they release their drug cargo within the low pH milieu. They have been specifically designed for targeted delivery to the cancerous cells. *In vivo* studies have shown the potential of the new material to inhibit tumor formation and reduce intraperitoneal adhesions without any significant adverse effects (195).

In view of the ease of transport of cancer cells through the blood-brain barrier (BBB) and interaction with other cells, pH-sensitive, Glioblastoma (GBM)-cell membrane-camouflaged biomimetic nanoparticles (MNPs) have been developed. According to the results of the experiments conducted, it can be argued that MNPs could efficiently carry TMZ and CDDP drugs through the BBB and deliver them to the GBM. The application of a pH-responsive dextran-based copolymer significantly increased the efficiency of combination therapy (196).

### 6.2 Nanocarriers for reprogramming the TME

As the tumors grow through their life span, they establish unique microenvironments different from other body environments; these environments significantly affect the development of tumors as well as response to therapies (197, 198). The ECM, immune cells, and blood vessels form part of various components of the tumor microenvironment that is defined as the immediate environment of tumor cells. Characteristics of tumor microenvironment include hypoxia, low acidity, aberrant vascularization, and abundance of immunosuppressive cells in the tumor microenvironment (199).

In essence, the TME is made up mostly of immunosuppressive cells such as TAMs, CAFs, MDSCs, and Tregs (200). Not only do TMEs help in tumor angiogenesis and metastasis, but TME can also lead to treatment failure and resistance (201). As a result, TMEs have become a target for cancer therapy, and many immunotherapy drug delivery systems use the TMEs as stimuli for controlled release of drugs (198).

Effective programming of TME and immunosuppressive state reversal can lead to the improvement of tumor immunotherapy. Modification of TME and reversing the immunosuppressive effect of immunotherapy will allow scientists to enhance the efficacy of the therapy (202). The failure of drug targeting leads to negative outcomes and decreases therapeutic value; however, the use of nanodrugs brings about the advantages of improved bioavailability, tumor-targeted drug delivery, and lower toxicity (203). Nanodrugs as drug delivery vehicles are capable of regulating the TME and carrying different drugs with various sizes that help reduce the immunosuppressive effects on immune cells in the TME (204). Despite notable developments in developing methods of TME treatment, there still remain some issues including low efficiency and toxicity of the developed strategies (205).

Moreover, a single drug is usually not sufficient to produce a proper anticancer immune response due to the complexity of immunological aspects of the tumor microenvironment. Thus, multiple-drug approaches and targeting multiple mechanisms are needed to change the TME and boost cancer immunotherapy. Different nanodrugs are recently created to regulate TME and modulate the immune response in order to detect and cure cancers (202).

Nanocarriers that are able to reverse immunosuppression in the tumor microenvironment are liposomes, polymeric nanoparticles, metal nanoparticles, and multifunctional nanoparticles. For example, in a study, liposomes that included a matrix metalloproteinase (MMP) and PD-L1 inhibitor covalently bonded with low concentration Adriamycin revealed enhanced antitumor effects (95).

Metal nanoparticles can generate local heating within the tumor tissues to improve the therapeutic efficacy of the immune system. For instance, in a study, Chen et al. constructed a multifunctional nanosystem based on metal particles (FA-CD@PP-CpG). It promotes the infiltration of CTL and suppresses the immune-suppressing cell populations, leading to improved anticancer therapy along with immunotherapy using photothermal therapy to reduce the tumor burden (206).

TAMs have been identified as a possible target for cancer immunotherapy. An innovative molecular-targeting strategy was established using the α-peptide and M2pep (M2 macrophage binding peptide) controlling M2-like TAM dual-targeting nanoparticles (M2NPs). The incorporation of anti-CSF-1R small interfering RNA (siRNA), which disrupts the survival signaling of M2-like TAMs, results in elimination of M2-like TAMs from melanoma tumors. This method significantly decreases tumor mass, improves survival rate, and eliminates TAMs (207).

A novel nanotechnology (RBC-Fn-NP) has been created to address the stromal obstacle of PDAC that hinders the delivery of medicines. In the novel strategy, RBC-derived vesicles are utilized to offer some form of protection. Targeting the ECM components (collagen I, fibronectin), and cancer-associated fibroblasts (CAFs) using FnBPA5 peptide is included. Additionally, RA inhibits the formation of ECM proteins by targeting the Golgi apparatus of CAFs. The consequence of the above is improved drug penetration, TME alteration, and finally increased chemotherapy efficacy (208).

Through CeMOF’s lactate oxidase and glutaminase inhibitor (CB839) reloading, Feng et al. designed a novel nanoplatform (CLCeMOF). Through blocking glutamine metabolism, the nanoplatform reduces the amount of immune-suppressive cells, including Tregs, MDSCs, and M2-TAMs. In addition to the lactate oxidase, the above nanoplatform aids in neutralizing the unfavorable tumor microenvironment, hence increasing immunotherapy efficiency (209).

### 6.3 Normalizing the tumor vasculature using nanocarriers

Another prominent characteristic of solid cancers is the presence of neovascularization, which may occur under hypoxic conditions in addition to TME. They contribute to the development of highly permeable vessels in the TME and provide various nutrients to support continuous tumor growth (210).

The reasons for aberrant angiogenesis in tumors include: (1) angiogenic factors, such as vascular endothelial growth factor (VEGF) and angiopoietin (ANG2); (2) proteins involved in pericyte recruitment and vascular maturation, including RGS5 and platelet-derived growth factor B (PDGFB); (3) proangiogenic processes assisted by tumor-associated macrophages (TAM), and (4) regulation of key angiogenesis regulators leading to vascular dysregulation in tumors, especially prolyl hydroxylase domain protein 2 (PHD2) (211).

Furthermore, aberrant blood vessels hinder perfusion, resulting in greater tumor hypoxia and acidity, thus stimulating tumor development (212). The aberrant blood vessel network inhibits the entry of immune cells into tumor tissue through blood vessels, thereby reducing immune cell concentration in the TME, leading to an ineffective immune response against the tumor (213). Therefore, controlling tumor blood vessels could be used to combat immunosuppressive conditions and restrict tumor material and energy transport necessary for growth, improving cancer immunotherapy (214). Studies have shown that anti-VEGF drugs at adequate dosages can not only correct the blood vessel structure but also reverse the immunosuppressive properties of the tumor microenvironment, making the vaccine-based anticancer immunotherapy more effective (215).

An investigation developed an injectable nanosystem incorporating PLGA microspheres, anti-VEGF, and a hydrogel composed of poly(N-isopropyl acrylamide) to treat anticancer angiogenesis. From the study findings, mice treated with anti-VEGF-loaded nanoparticles (60%) exhibited a significantly smaller lesion size than the untreated control group of the study (216).

The use of nanomedicine with the EGFR inhibitor erlotinib in a CT26 colon cancer mouse model may enhance drug delivery, reduce immunosuppression, and promote blood perfusion and oxygenation (217). To increase the efficacy of treatments through positive feedback regulation of vascular normalization and tumor microenvironment modulation, Deng et al. developed two nanotherapies, MAR/MPA and FLG, in order to target the VEGF/VEGFR vascular healing signaling pathway and block the CCL5/CCR5 signaling pathway, respectively (218).

Nitric oxide (NO) plays a crucial role in regulating angiogenesis and maintaining vascular stability. Nevertheless, currently, there are no long-term delivery systems that offer extended release. Scientists have developed a nanotherapeutic drug, NanoNO, for sustained delivery of nanoscale nitric oxide (NO) release in liver cancer. With the use of a small amount of NanoNO, tumor vessels can be normalized; additionally, it promotes drug and ligand delivery, as well as reprogramming of the tumor microenvironment from immunosuppressive to immunostimulatory. This leads to increased efficacy of not only chemotherapeutics but also cancer vaccines, overcoming drug resistance (219).

Also, another research indicated that two-dimensional ZnFe(CN)_5_NO nanosheets could be generated using Zn²^+^ and sodium nitroprusside, a widely used antihypertensive drug. However, under certain conditions, the nanosheets are able to release NO and, therefore, improve tumor immunotherapy through the reversal of the suppressive tumor microenvironment (220).

A new kind of peptide amphiphile nanoparticles has been developed for use in immunotherapy and tumor vascular normalization therapy. In particular, it comprises an immune checkpoint inhibitors peptide and antiangiogenic peptides. The outcomes suggest that the nanoparticle encourages immune cells infiltration and restores antitumor immunity, thus providing a promising nanomedicine approach to improve cancer immunotherapy (221).

### 6.4 Modulation of tumor hypoxia for better drug delivery

Hypoxia is one of the characteristics of solid tumors as a result of the mismatch of tumor growth requirements for nutrients and vascularization of the solid tumor mass. It is one of the causes of tumor aggression and poor prognosis in many cancers (222). Hypoxia leads to a chain of reactions in which signaling pathways related to survival are activated. The stability of hypoxia-inducing factors contributes to the expression of genes involved in cell proliferation, metabolic processes, anti-apoptosis, angiogenesis, and epithelial mesenchymal transformation (223, 224).

The role of hypoxia in tumor development and metastasis includes not only support for its propagation but also inhibition of the effectiveness of virtually all types of cancer therapy – chemotherapy, radiation therapy, phototherapy, and immunotherapy (225). This has led to the appearance of studies dedicated to hypoxia suppression methods to improve antitumor therapy. Oxygen supply enhancement, oxygen consumption suppression, and the prevention of the HIF signaling pathway are the three main directions of tumor hypoxia treatment (226).

Nevertheless, research has revealed that due to the dispersal of therapeutic agents in various parts of the body and their poor penetration into solid tumors, side effects and inefficacy limit the progress of existing techniques (227). Fortunately, through the solution of the above constraints, nano drug delivery systems (NDDS) have displayed promising potential in the reduction of tumor hypoxia, providing several benefits like deep penetration, tumor-targeted distribution, long-term intratumoral retention, and extended blood circulation (226).

Many nanodelivery technologies have been designed to target tumor hypoxia taking advantage of the features of NDDS (228). Nowadays, there are two main strategies for the fabrication of nanodelivery technologies to improve the oxygen content in tumor tissues: firstly, oxygen transport to oxygen-bearing nanodelivery systems within tumor tissues via blood substitutes such as hemoglobin and perfluorocarbons; secondly, drug delivery systems that facilitate the decomposition of hydrogen peroxide (H_2_O_2_) in tumors into oxygen, for instance, MnO_2_ or catalase (CAT) nanoparticles (229).

Several other O_2_ biomimetic nanocarriers have been synthesized by Chen et al. (230). Human hemoglobin was incorporated into self-delivering drug carriers comprising poly(lactic-co-glycolic acid) (PLGA), human serum albumin, and cancer cell membranes. This would enhance the antitumor effect. The nanocarrier system comprises mesoporous iron oxide nanoparticles with glucose oxidase and perfluorocarbon (PFC), a chemical compound that transports oxygen, loaded inside the nanoparticles. These were designed to look like cell membranes. Using photothermal therapy, chemokinesis, and fasting therapy, their combined approach will maximize the anticancer benefits (231).

Song et al. proposed a nanodelivery strategy that offers radiosensitization therapy and oxygenation of the tumor site. Hollow mesoporous inorganic PEGBi2SE3 nanoparticles can be used to deliver photosensitizer or anticancer drugs. Droplets of perfluorocarbon, a compound that carries oxygen, were infused into the nanoparticle chamber. When exposed to NIR irradiation, PFC releases oxygen that lowers hypoxia in TME, enhancing radiation efficiency (232).

Cheng et al. encapsulated catalase (CAT) in MOF-based nanodrugs delivery vectors containing either photosensitizers or anticancer drugs. *In vivo* oxygen supplementation via this delivery technique increases chemotherapy and PDT effectiveness by inhibiting rapid CAT degradation and promoting efficient tumor localization of the enzyme (233).

Certain inorganic molecules, such as manganese dioxide (MnO_2_), Prussian blue (Pb), and platinum (Pt), have been found to exhibit the features of catalase (CAT) and can catalyze the decomposition of hydrogen peroxide (H_2_O_2_) to oxygen (O_2_) efficiently (234). In the study conducted by Yang et al., an H-MnO_2_-based nanoplatform co-loaded with two drugs, namely doxorubicin (DOX) and chlorin e6 (Ce6), was developed. It was observed that the treatment with the above-mentioned nanoplatform helped alleviate the hypoxic state in the tumor. According to the experimental data obtained from the investigation, the abundance of O_2_ molecules generated from the abovementioned nanoplatform contributed significantly to the enhanced efficiency of chemotherapy and photodynamic therapy (PDT). Immune cell death (ICD) followed by PDT and chemotherapy helped stimulate the maturation process of dendritic cells (DC) and activation of T cells. Additionally, the M1-type TAMs replaced the M2-type TAMs (235).

### 6.5 TME immune cell modulation as a fundamental approach in nanomedicine

Tumor immune cells determine the immunological microenvironment of a tumor, dictate response to therapies, and significantly affect nanoparticle behavior. Despite their vital role, interactions between nanomaterials and immune cells are underrepresented in research compared to physical-chemical or regulatory perspectives (165). TAMs act as pivotal regulators of tumor development and therapy resistance; predominantly, they exhibit a phenotype resembling an immunosuppressive M2 macrophage, encouraging angiogenesis, ECM degradation, and metastasis. Nanomaterials can modify the macrophage profile by selectively administering compounds that either eliminate M2 macrophages or convert them to pro-inflammatory M1 macrophages. Liposomal clodronate, nanoparticles carrying siRNA, and TLR agonist nanoparticles have already proven effective in this process. In contrast to systemic macrophage elimination, targeting TAMs via nanotechnology allows for high spatial resolution, enabling tumor “re-immunization” without immune system compromise (236).

In addition to macrophages, dendritic cells (DCs) represent an essential cell type, controlling antigen presentation and T cell activation, while being profoundly inhibited by tumors. DCs could be stimulated using nanoparticles conjugated with antigens and adjuvants, allowing direct targeting and activation, irrespective of tumor-induced inhibition.

The use of polymeric nanoparticles to deliver CpG oligodeoxynucleotides and/or STING agonists has also led to excellent DC activation, thus increasing T cell infiltration and synergizing with checkpoint inhibitors. Similarly, myeloid derived suppressor cells (MDSCs) have a significant role to play as they inhibit T cell function, promote tumor progression, and hence are the focus of cancer-targeted nanomedicine (237).

Nanocarriers that are able to modulate chemokine signaling pathways like CXCR2 pathways have been found to cause considerable reduction in MDSC recruitment. This results in the establishment of a conducive environment for T cells. MDSCs cannot be easily targeted systemically without adverse side effects, hence the significance of using nanocarriers to achieve targeted inhibition (238).

It is worth noting that T cells continue being the main effector cells in tumor elimination, but they are subjected to tumor-induced immunosuppressive and metabolic conditions. Here, nanoparticles can be used to counter this exhaustion through localized delivery of cytokines like IL 12/IL 2 and consequently achieve excellent T cell proliferation (239).

Furthermore, nanoparticles can deliver metabolic adjuvants capable of restoring the health of T-cells within nutrient deprived, acidified, or hypoxic areas within the TME, which has gained recognition in reversing the suppression of T-cell functionality in resistant tumors (240). Another potential avenue of research involves the localized delivery of checkpoint inhibitor drugs through nanoparticles to improve intratumoral concentrations without systemic side effects.

NK cells, although typically not part of the TME discussion, represent innate killers that are often suppressed within many solid cancers. Nanoparticles delivering IL-15 or its receptors would allow for the restoration of NK cells, providing advantages in cancers with minimal T cell infiltration. Fibroblasts and stromal cells, on the other hand, indirectly affect the success of the immune system by acting as a barrier for immune infiltration (241).

Finally, nanoparticles containing enzymes to degrade the ECM such as collagenase or hyaluronidase could help remodel the structure to allow easier infiltration. In addition, increased T cell entry into tumors by manipulation of ECM components is especially relevant in “immune excluded” cancers where T cells tend to be retained at the tumor’s periphery. Cytokine reprogramming represents another crucial aspect of nanoparticle technology, since by manipulating cytokine gradients such as IL 10, TGF β, or GM CSF, nanoparticles may transform the immune suppressive TME into an immunostimulatory one (242).

Cytokine reprogramming may further potentiate immunotherapy efficacy by making the TME more amenable to immunological processes. Moreover, nanoparticles can be used as carriers for achieving ICD, leading to enhanced antigen presentation and priming of T cells. Coupling ICD generation and immune reprogramming nanocarriers can create a positive feedback loop involving antigen exposure and immune stimulation (243). In particular, immune modulation is beneficial for targeting tumor heterogeneity since immune signaling pathways are more conserved between patients compared to the inherent biological properties of cancer cells. The predictability of immune targeting nanomedicine is thus higher than physicochemical TME properties such as pH or permeability of the blood vessels. Regulatory bodies prefer approaches with mechanistic understanding and immune modulating nanoparticles allow quantifiable mechanistic endpoints such as polarization of TAM or T cell proliferation. Additionally, AI algorithms can predict the dynamics of immune cells better than other aspects of the TME as high-quality immunological data sets at single-cell level become increasingly accessible (40, 237, 240).

Combining the immune modulation of TME cells with AI prediction and particle targeting creates an integrated approach to nanomedicine. In conclusion, concentrating on TME immune cell modulation instead of a broad range of different fields contributes to the cohesion of the manuscript and addresses the reviewers’ comments (37, 240).

## 7 Preclinical and clinical applications of TME-targeted nanomedicine

### 7.1 Preclinical models for testing nanocarriers for targeting TME

**Figure 8:**
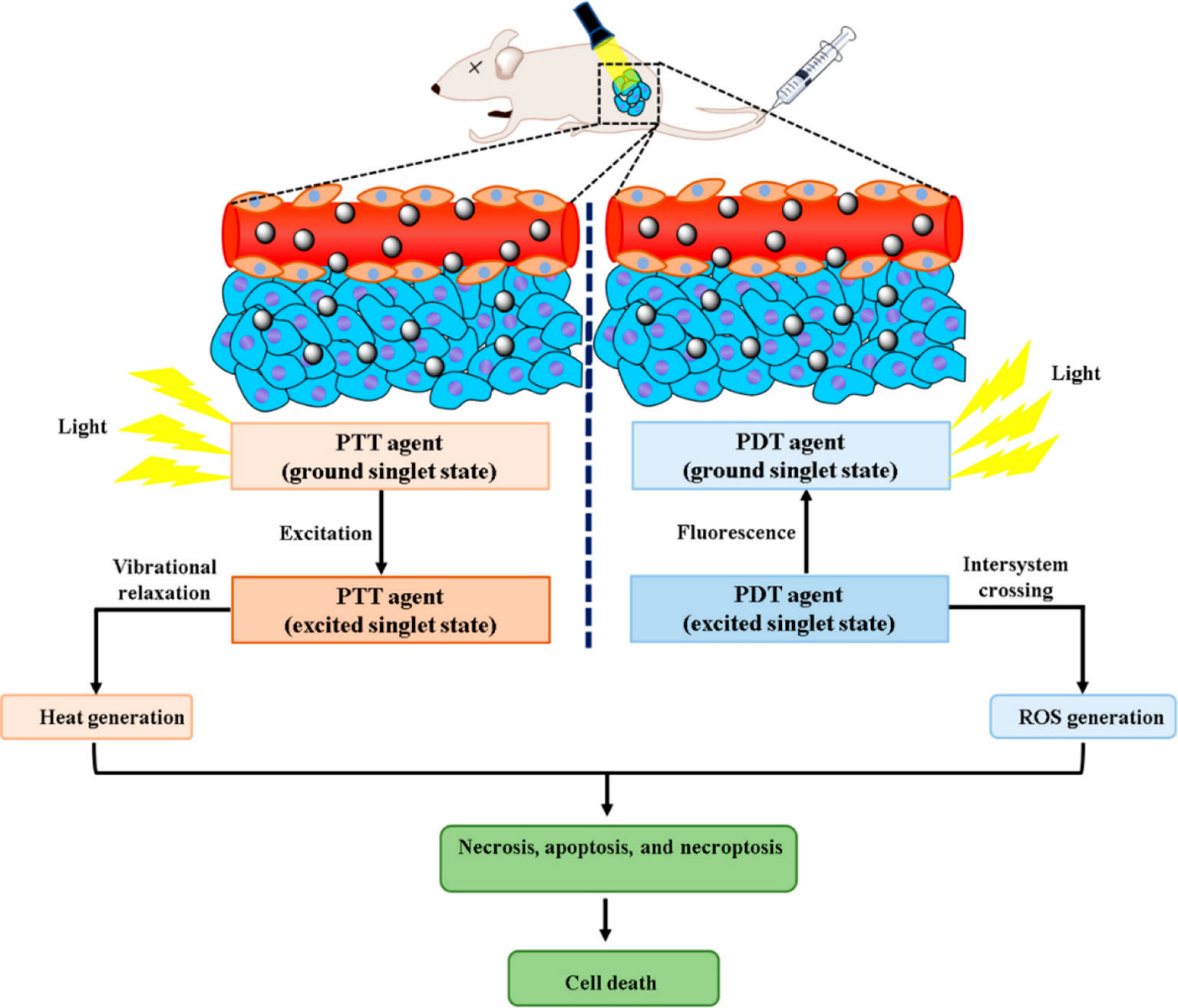
The above figure explains the process of killing cancer cells through cancer nanomedicine in PTT and PDT (245).

The design of nanocarriers that target TME requires thorough preclinical investigations using models that accurately reflect the very complex structure of TME. New preclinical models are highly valuable tools for assessing the effectiveness of nanocarriers and predicting clinical results (244). **Figure 8** shows the concept of cancer nanomedicine capable of destroying cancer cells through PTT and PDT methods. *In vitro* models have developed from simple 2D cell cultures to more advanced 3D systems (246). Spheroid and organoid cancer cells are able to better mimic solid tumors because of their complexity and structural diversity; hence, they can be used as models for testing new TME-targeting nanocarriers (246, 247).

Such preclinical models were proved to be effective in assessing nanocarrier penetration and drug delivery kinetics under conditions similar to those of tumor microenvironment (248).

**Figure 9:**
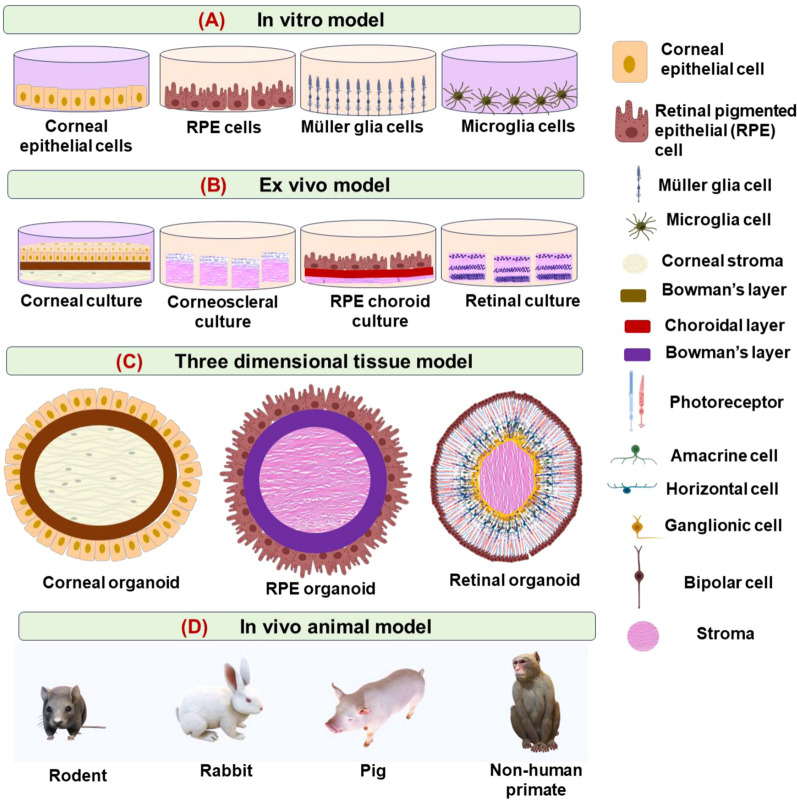
Schematic representation of pre-clinical disease model of eye disease. **(A)** For assessing the efficacy, toxicity, and bioactivity of nanomedicines and drugs, *in vitro* 2D culture of ocular cells is used. **(B)** To determine the formulations of drugs and nanomedicines and optimizing their concentrations, ex vivo cultures of ocular tissues are used. **(C)** In preparation for animal studies, three-dimensional ocular tissue culture models like organoids are used to assess drugs and nanomedicines. **(D)** *In vivo* analysis of drug delivery systems and nanomedicine is conducted using animal models (250).

Animal models play an important role in a thorough analysis of nanocarriers designed to target the TME. Patient-derived xenografts (PDX) can be considered an appropriate model, since such an approach allows maintaining intact characteristics of the original tumors and obtaining relevant information on the behavior of nanocarriers in a complicated biological system (248). The use of syngeneic mice models with functional immune systems is also critical when analyzing the impact of nanocarriers on immunological mechanisms of the TME (249). A hierarchical scheme of analysis of nanomedicine and drug candidates for eye-related pathologies is outlined in **Figure 9** below.

### 7.2 Clinical translation of TME-targeted nanocarriers

TME-specific delivery systems have seen various successes and setbacks as they move from the bench to bedside. A number of novel nanotechnology-based approaches to target components of the tumor microenvironment have reached clinical trials, some of which have been approved by the FDA. Significant breakthroughs such as the development of Abraxane and DOXIL showed that EPR-mediated transport and intrinsic pathways could be harnessed to restore efficacy of conventional chemotherapy drugs (251–254).

Current studies have focused on implementing more effective methods of delivery systems targeting tumors. A phase I study of anti-MUC1 peptide-coated nanoparticles loaded with paclitaxel resulted in favorable effects in patients with drug resistant breast cancer, which were due to enhanced intratumoral drug distribution and reduced toxicity (255). Nevertheless, problems continue to arise in translating these results into consistent clinical outcomes, especially in terms of process development and reproducibility (256).

The first nanomedicines paved the way for advanced nanotechnology platforms capable of actively responding to the presence of TME-related stimuli. Further clinical research has focused on nanodelivery vehicles that utilize the distinctive biological signals from the TME. The hypoxia-activated prodrug evofosfamide (TH-302) has been extensively evaluated in various solid tumors including pancreatic cancer and soft tissue sarcoma in clinical trials. Even though it is not a nano-platform, it demonstrates the clinical approach of hypoxia-directed methodologies, thus proving the concept of bioreductive drugs that could selectively target hypoxic tumor regions (257–259).

In the same fashion, other therapies utilizing TME elements have also been investigated. The conjugated molecule vintafolide, which consists of a folate-targeted ligand and a vinca alkaloid drug, tries to take advantage of the overexpression of folate receptors in specific cancers, reaching Phase II clinical trials (260, 261).

**Figure 10:**
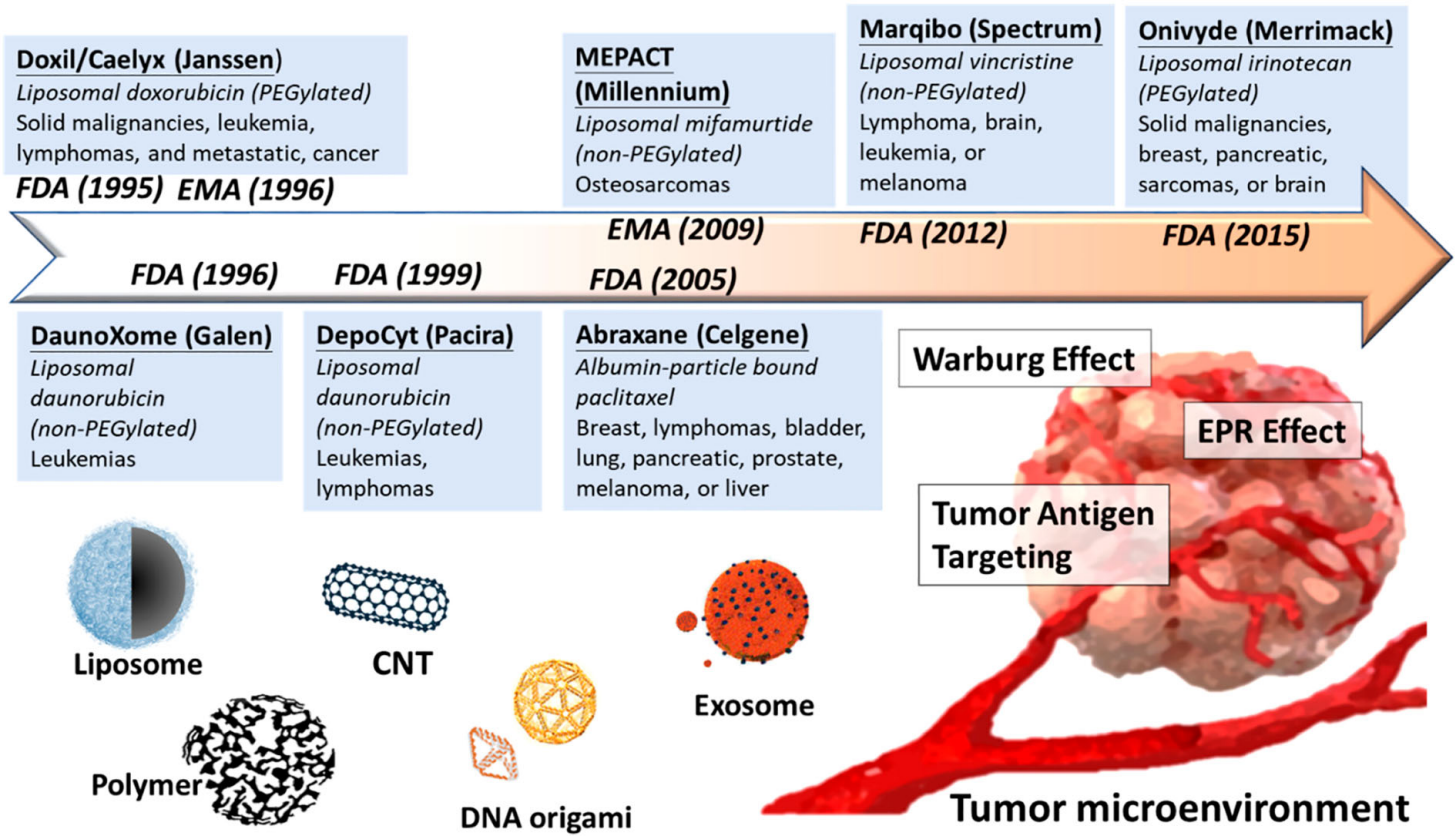
Tumor microenvironment targeting is one of the directions of nanomedicine development. The development of numerous nanocarriers has moved from pre-clinical testing to clinical evaluation and, finally, to approval for use in cancer therapy over the past decades. The action of both active and passive delivery approaches can facilitate entry into the tumor microenvironment. Active delivery is associated with direct recognition of tumor antigens via conjugation with high affinity molecules, while passive delivery relies upon low pH and permeability of the tumor vasculature (Warburg effect and EPR effect). Novel and highly developed nanocarrier materials include liposomes, polymers, carbon nanotubes, DNA origami, and exosomes (263).

Moreover, there have been efforts in designing stimulus-responsive drug delivery systems, such as ThermoDox, a thermosensitive liposomal delivery system of doxorubicin, in combination with radiofrequency ablation (RFA). The heat generated by RFA leads to rapid drug release within the tumor tissue, thus representing an example of an experimentally validated system for controlled release and drug delivery using an external stimulus to the TME (262). **Figure 10** shows how nanotechnology has been applied for targeting the TME through various stages of development of nanocarriers from preclinical research to clinical applications in cancer therapy.

### 7.3 Safety, toxicity, and pharmacokinetics of TME-targeted nanocarriers

It is crucial to keep in mind the safety concerns when designing nanocarriers for targeting TME. It would be useful to conduct a comprehensive analysis regarding not only acute and chronic effects but also immunogenicity and inflammation (264). The significance of knowing protein corona formation and its effects on the fate of nanoparticles *in vivo* has been recently highlighted (265–267).

**Figure 11:**
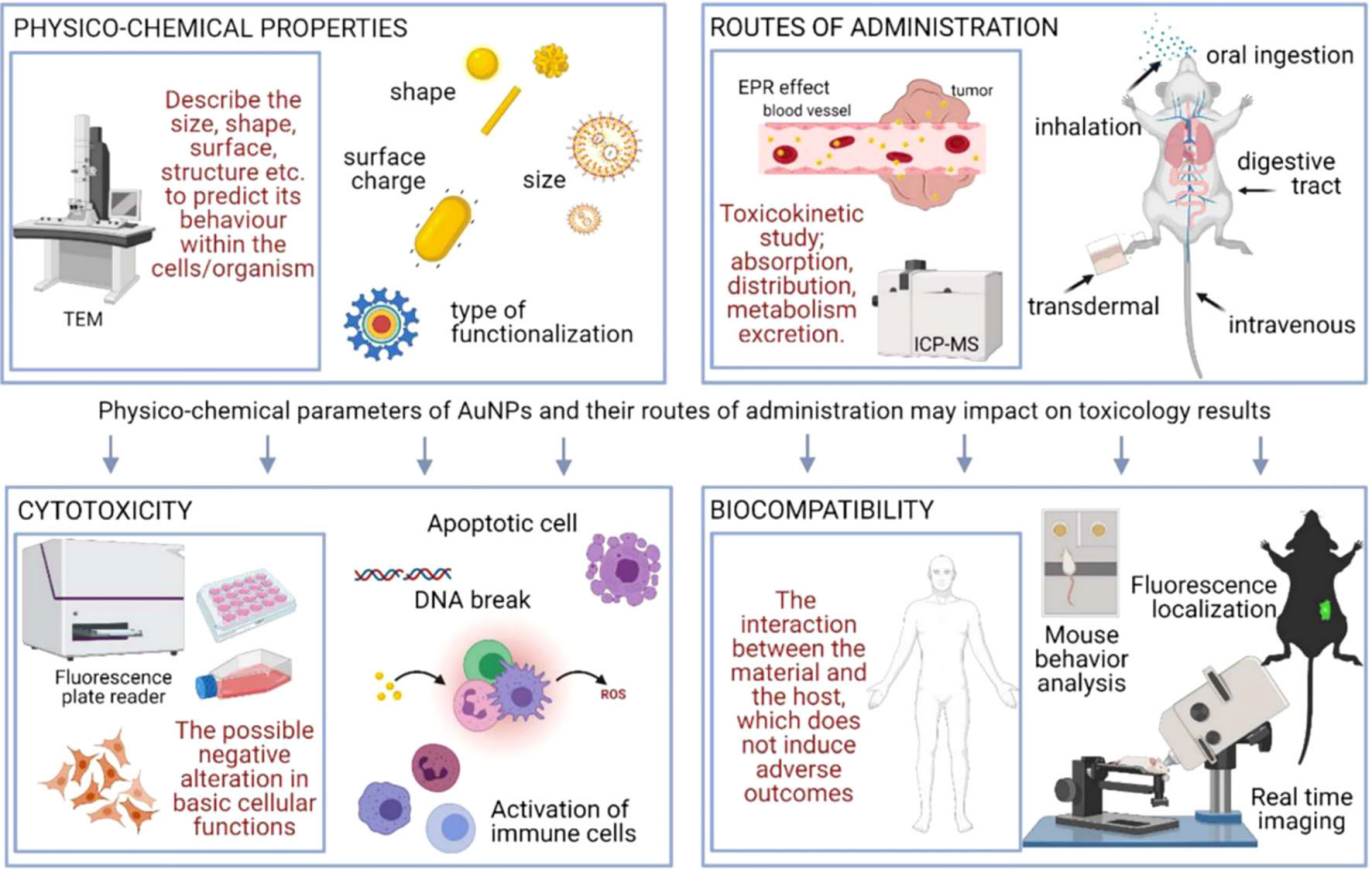
Outcomes related to safety and toxicity, such as negative effects (apoptosis, increased production of reactive oxygen species, alterations in behavior of animals, etc.), are highly controlled by various factors associated with aspects influencing biocompatibility and safety of nanoparticles. Such aspects include particle size/hydrodynamic diameters, morphology, nanoparticle shape and surface, as well as modes of administration of nanoparticles in patients (270).

TME-targeting nanocarriers tend to demonstrate significantly different pharmacokinetics compared to their conventional counterparts. The complex mechanisms behind the process were revealed using advanced imaging technologies, thus calling for careful tuning of the carriers’ parameters (268). Accumulation in some organs such as liver and spleen was noted during longitudinal experiments, which brings concerns regarding potential toxicity (269). **Figure 11** gives an example of nanoparticle-based safety and toxicology depending on design and delivery parameters.

## 8 Emerging strategies in TME-targeted nanomedicine

### 8.1 Smart nanocarriers: real-time sensing and drug release

**Figure 12:**
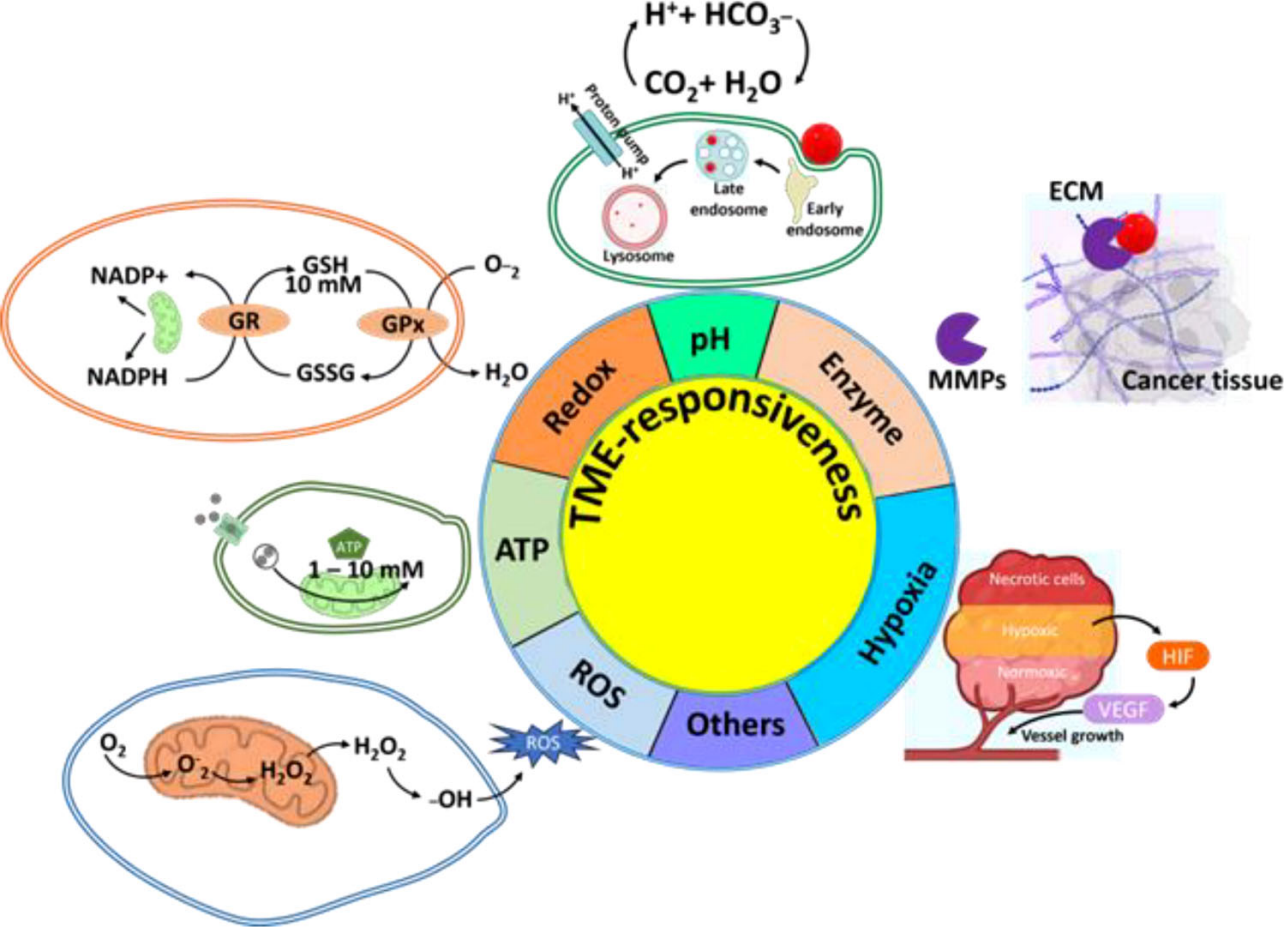
Diagrammatic illustration of TME response for polymeric micelle-based drug delivery (272).

The introduction of smart nanocarriers represents a significant advancement in cancer treatment through targeted therapies, utilizing intelligent delivery systems that can respond to specific signals from the tumor microenvironment (TME) (271). Smart pH-responsive nanocarriers take advantage of the acid condition of TME, having a pH ranging from 6.5 to 6.8, by employing materials, capable of structural modifications or bond dissociations under such conditions. Studies have shown that polymeric micelles with pH-sensitive bonds released the cargo drugs more efficiently at low pH of TME than in blood circulation (**Figure 12**) (273–275). Enzyme-sensitive nanocarriers offer a targeted approach that is sensitive to enzymes being overexpressed in tumors like the case of matrix metalloproteinases (MMP). Such a system uses specific peptide sequences that will be cleaved enzymatically to release the drug (276, 277).

The redox-based systems offer a potential strategy for exploiting the enhanced concentration of glutathione in tumor tissue. The disulfide bond in such a system allows the system to be stable until in comes into contact with cancer cells where it is rapidly cleaved due to the reduced state of the cell. This strategy has proven to be highly efficient in delivering hydrophobic drugs (278, 279).

### 8.2 Major areas of knowledge gap in cancer biology and nanoparticles

Though nanomedicine has progressed significantly in recent years, there are many biological pathways that play critical roles in nanoparticle behavior which we know very little about. In turn, our inability to predictively design nanoparticles has led to inconsistent therapeutic performance across various tumors and patients. For example, the biology behind how the protein corona forms and changes its structure has not yet been clearly elucidated. We can measure what proteins make up the corona, but what determines selective protein binding and how slight modifications in the corona will impact cell recognition or uptake is yet to be determined.

Furthermore, the pathway that dictates nanoparticle transport through the MPS is still not completely understood. Why do certain nanoparticle formulations show drastically different uptake by macrophages? (280).

Lastly, one of the key unresolved issues lies in understanding tumor penetration. Though EPR has been well researched in the last few decades, the biology behind the variability of the EPR effect between different tumor models or even patients, is poorly characterized.

The other important topic that requires detailed biological exploration pertains to endosomal escape. There is no definitive model which describes the mechanism by which different nanocarrier chemistries disrupt endosomal membranes, or even how effective this disruption is for different cell types and in either hypoxic or normoxic regions within tumors. The interaction with the ECM needs further exploration too. For example, the effect of ECM stiffness, cross-linking and glycosaminoglycans composition on nanoparticle transport is poorly understood and is largely empirical (32, 158).

Furthermore, the immunological impact of repeated administration of nanoparticles remains unclear. Though there are many experimental works on one time administrations of nanoparticles, the characterization of adaptive immunity, including immunological memory and nanoparticle specific tolerance is still not well-developed (281). Tumor-associated macrophages, dendritic cells, myeloid-derived suppressor cells and fibroblast populations demonstrate heterogeneity that creates great variability in nanoparticle behavior. However, data quantifying the nanoparticle-physiology relationships are still poor. Yet another gap lies in the incomplete characterization of the routes used in the intracellular trafficking of nanoparticles. Not all nanoparticles undergo classical endocytic trafficking processes; there are those that travel through nonclassical routes. What determines these nonstandard trafficking mechanisms is not known. Further work is also needed regarding biological barriers at the organismic level. Studies on nanoparticle trafficking and clearance have been largely conducted on rodents, while translating these to human systems remains a challenge due to physiological differences (282).

Comorbidities affecting the patients could also affect nanoparticle functionality, and how this happens is currently not known. Diabetes mellitus, chronic inflammation, or changes in lipid metabolism can greatly alter nanoparticle trafficking, but research is limited in this regard. The microbiota-nanoparticle interface is an entire field waiting to be explored. Current research points to possible modification of nanoparticle surfaces by microbiota in the gut and tumors or metabolism of nanoparticle drugs, among other phenomena (283).

However, even within the tumor itself, intratumoral heterogeneity still stands as a biological mystery. Parameters such as pH, oxygen levels, vascularization, metabolic conditions, and the presence of immune cells all play an important role in how nanoparticles function, although there are very few scientific investigations done on such parameters in terms of their spatial and temporal gradients. All these biological uncertainties underscore the necessity of more mechanistic research into immunology, tumor biology, systems pharmacology, and high-resolution imaging techniques (281, 282).

### 8.3 Designing nanocarriers using artificial intelligence

**Figure 13:**
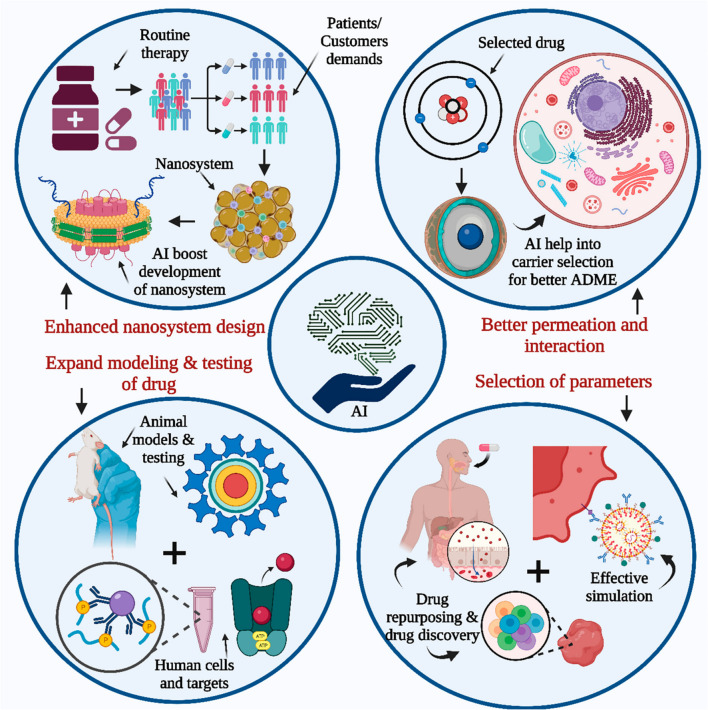
Application of AI in developing new medications. The existing system for modeling drug trials can be enhanced, the nanosystem design can be optimized, and the parameters and factors considered in designing new drugs, drug discovery, and drug repositioning methods can be optimized using AI. The study of the permeability of drugs, simulation of the drug, human cell receptors, etc., will help to understand the membrane interaction mechanism with the human body model (286).

In targeted cancer therapy, studies incorporating artificial intelligence (AI) into the design of nanocarriers have been conducted extensively. Machine learning has proven successful in predicting some crucial parameters of nanocarriers, such as the effectiveness of drugs loaded into carriers, release kinetics, and biodistribution (284, 285). **Figure 13** demonstrates how AI assists in each step of contemporary drug development processes.

Machine learning algorithms are promising in exploring intricate relationships between structures and activities of nanocarriers. Shin et al. utilized neural networks for better surface modification of nanoparticles, leading to increased efficacy in tumor targeting (285, 287).

### 8.4 Validated machine-learning architectures for predicting nanocarrier behavior

Although early discussions of AI-driven nanocarrier design were largely theoretical, several machine-learning architectures have now demonstrated reproducible, experimentally validated predictions for nanoparticle behavior. This enables predictions of physicochemical properties, biological interactions, and *in vivo* efficacy; thus, significantly minimizing trial-and-error efforts in synthesis (288, 289).

Amongst the most early and validated model architectures is the random forest (RF). RFs perform excellently in predicting nanoparticle cytotoxicity, cellular uptake, and hydrodynamic size following formulation, as supported by validation through TEM, DLS, and flow cytometry measurements (290).Another well-validated model architecture that has achieved great success includes support vector machine (SVMs). They are known to classify nanocarriers based on their hemolytic activity, macrophage uptake tendency, and passive tumor accumulation efficacy. High dimensional physicochemical parameters facilitate SVM prediction of complicated biological outcomes with minimal experimental datasets (291, 292). Models that have performed exceptionally well include the gradient boosting techniques – specifically, XGBoost and LightGBM. The main advantage is that they are highly effective in modeling nonlinearity in the relationship between polymer composition, molecular weight distribution, and pH and ionic strength (293).

Predicting the drug loading capacity, encapsulation efficiency, and degradation rates of PLGA and lipid nanoparticles have been successfully achieved through artificial neural networks (ANNs), specifically feed forward multilayer networks. Validation is usually done using HPLC-based quantification of loading and release compared to ANN results (294). For more complicated structures, like polymeric micelles, dendrimers, and multiple-layered hybrid particles, graph neural networks (GNNs) have been utilized due to their higher computational power. Validations have been performed for predicting dendrimer–protein interactions, ligand spatial distribution impact on activity, and endosomal escape structural factors (295). Message passing neural networks (MPNNs) offer improved prediction performance by including connectivity data at the atom or monomer levels. Validations have been conducted using MPNN models for predicting the composition of the protein corona where the predicted proteins matched the mass spectrometry protein panel data (296). Convolutions neural networks (CNNs) enable image-based prediction models to be made. Validated applications using CNN include predicting nanoparticle aggregation from TEM images, classifying lipid nanoparticle morphologies, and quantifying structural heterogeneity without any need for manual feature extraction (297).

Transformers, a recently developed family of architectures that is adept at handling multimodal input streams like structural features, spectral fingerprints, and biochemical measurements, have been successfully implemented to predict variability in circulation half-life and tumor uptake in animal studies. Transformer-based architectures can perform tasks like sequence prediction and hence estimate conditions-dependent performance, such as pH-induced release and enzymatic breakdown, with dynamic release assays employed to validate their predictive power (298).

Latent models based on autoencoders have been utilized for mapping high-dimensional design spaces to simpler lower-dimensional ones. Experimental results indicate that interpolated nanoparticles between latent designs usually exhibit consistent transition in loading capacity and release behavior. Novel polymer structures generated by variational autoencoders (VAE) and generative adversarial networks (GAN), with experimentally verified biodegradation and physical properties for controlled release, have been reported (299, 300).

Approaches employing reinforcement learning (RL) techniques for optimizing nanoparticle formulation by interacting with predictive simulators have been validated by conducting experiments with synthesized recommended structures and comparing them against manually designed baseline formulations. However, for this kind of approach to be effective, high-quality sets of physicochemical descriptors are required. Among them can be listed particle size distribution, surface charge, core-shell composition, hydrophobicity, ligand density, polydispersity, and mechanical properties (301, 302).

As well as it, models need *in vitro* biointeraction descriptors, including such parameters as cellular uptake kinetics, lysosomal traffic, cytotoxicity, and stability. Flow cytometry, confocal microscopy, and live-cell techniques are the major sources of this type of data. In turn, accurate prediction of *in vivo* biocompatibility of nanoparticles necessitates biodistribution descriptors, which could be obtained via radiolabeling, fluorescence microscopy, or ICP MS. Such descriptors allow linking design parameters with tissue accumulation profiles (303, 304).

For the purposes of immunological prediction, one requires models utilizing immune responses, such as cytokine secretion, complement activation, macrophages polarization, and dendritic cells maturation. As in previous cases, such descriptors provide necessary information for making assumptions about the immunogenicity of nanoparticles and their ability to function as an adjuvant. For corona-related models, datasets of proteins interacting with nanoparticles, which can be obtained using LC MS/MS, will be sufficient.

Environment-sensitive release characteristics of nanoparticle models have to include datasets of pH-sensitive release patterns, enzyme-responsive particle breakdown, and redox-active drugs release profiles. This type of data allows us to establish factual relationships between structure, stimuli, and response (305). A combination of advanced machine learning algorithms based on proven physicochemical, biological, and *in vivo* datasets allows researchers to move from purely theoretical approaches in AI-based nanocarrier designs to reliable methods backed up by experimental facts. Predictive algorithms make it possible to engineer nanomedicines faster and more efficiently than via conventional experimental trial-and-error methods (306).

### 8.5 Combination nanomedicine with other therapies

**Figure 14:**
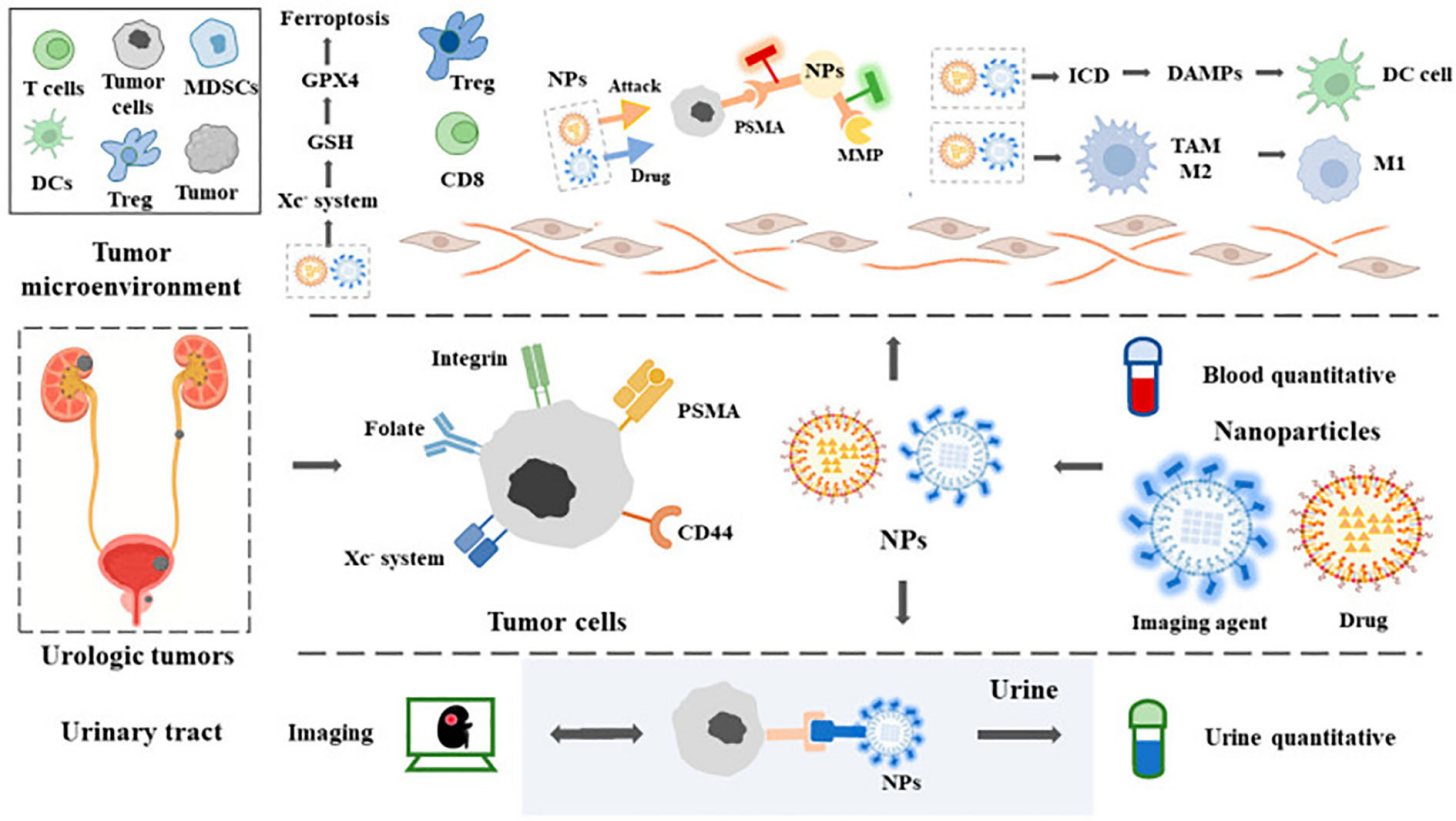
Diagrammatic representation of nanomedicine-based urologic tumor imaging, diagnosis, and treatment (307).

The combination of nanocarriers with various therapies has led to innovative solutions to problems related to drug resistance and effectiveness enhancement. It is clear from **Figure 14** that taking advantage of unique characteristics of nanocarriers, we could increase the efficiency of traditional treatment significantly and reduce the negative impact on patients’ health (131).

Nanocarriers exhibit remarkable potential in radiosensitization in conjunction with radiotherapy (308).For instance, gold nanoparticles enhance the effect of radiation through dose augmentation in specific areas. Recently, clinical studies have indicated the prospects of defeating cancer resistance through the combination of nanoparticle-mediated drug delivery and radiation treatment (308, 309).

The integration of nanomedicine with immunotherapy presents a fascinating possibility. Nanocarriers can administer immune checkpoint inhibitors effectively and at the same time regulate the immunosuppressive environment of the tumor. Such a method holds promise in increasing the effectiveness of immunotherapy and circumventing the drug resistance encountered with conventional immunotherapy (310, 311).Gene-editing applications, specifically those employing CRISPR-Cas9, have been significantly enhanced by the use of delivery systems based on nanocarriers. This helps protect the gene cargo in circulation and facilitates its uptake by cells (312). New developments in the use of lipid nanoparticles in gene delivery show promising results, particularly in addressing delivery efficacy and specificity (313–315).

## 9 Challenges and future prospects

### 9.1 Challenges in nanocarrier delivery to solid tumors

Despite significant progress in developing nanocarriers responsive to various tumor microenvironment (TME) cues, translating these systems into clinical practice for solid tumors remains a major challenge. Promising results observed in preclinical studies are rarely replicated in patients, largely due to the complex nature of tumors. One key obstacle is tumor heterogeneity, which exists at multiple levels (316). Inter-tumor heterogeneity implies that the TME varies considerably in terms of each type of cancer. Thus, the desmoplastic and hypovascular nature of pancreatic cancer microenvironment poses a bigger problem compared to less heterogeneous types of cancer and makes the development of an optimal carrier impossible (317).

Moreover, the intra-tumor heterogeneity ensures that the microenvironment is not homogeneous inside a tumor. Nanoparticles tend to concentrate in the highly perfused perivascular area around the tumor’s surface but fail to reach the poorly vascularized, hypoxic, and nutrient-starved core of the tumor, where high interstitial fluid pressure inhibits penetration. Thus, there will always be some cancerous cells inside the tumor left alive to cause relapse in the future. This spatial difference between the tumor’s outer layer and its core is also the reason for the inconsistency of the EPR effect, as it cannot be guaranteed that there would be enough pores in the vasculature, sufficient permeability of the blood vessels, and low intratumor fluid pressure. However, the main challenge remains the inconsistency of the TME from one patient to another. People with the same disease might have completely different tumor microenvironments, determined by their unique genetics, immunity, and stage of the disease (318). A drug delivery system targeting a particular acidity level or concentration of an enzyme could work effectively on one patient but show no result in another whose tumor microenvironment does not have such conditions (319).

Poor permeation is closely linked with heterogeneity. First, because of the dense ECM and high IFP, there is a serious physical limitation that impedes the ability of nanocarriers to move away from the proximity of blood vessels and reach deeper parts of tissues, resulting in a superficial and patchy drug distribution. Second, even active targeted nanoparticles might face a “binding site barrier”: they become bound with the first available target cells that appear along their path of migration, preventing any further penetration into the tumor mass. Thus, while larger nanoparticles have long circulation times, their penetration capabilities are poor; in contrast, smaller nanoparticles have greater penetration abilities but get eliminated faster. As such, this is a dilemma that cannot be easily solved in terms of fundamental nanoparticle design (320). Therefore, in conclusion, the combination of intra- and intertumoral heterogeneity, poor penetration across biological barriers, and the highly unpredictable nature of the TME makes up for the main obstacle on the way to effective utilization of nanomedicine to treat solid tumors.

### 9.2 Regulatory and manufacturing challenges

Translational problems associated with moving from effective preclinical nanomedicines to clinically approved medicines can pose considerable challenges in terms of regulation and production. Although various novel nanocarriers prove highly effective when tested in the laboratory, scaling up production for their clinical application poses serious challenges because of the complicated nature of the regulatory process (321). Indeed, one of the critical issues is related to manufacture and scale-up. Reproducing multifunctional nanocarriers in large quantities and ensuring GMP adherence may pose certain difficulties because of their complicated manufacturing process with several phases. Because any alteration in the manufacturing process of the nanocarrier can result in variation in the nanoparticle’s physical and chemical properties – size, charge, drug loading capacity, and ligand density, it is critical to address the issue of batch consistency, which is quite a challenge. Variation in the physical and chemical properties will have a drastic effect on how the nanoparticles behave within the body – pharmacokinetics, biodistribution, effectiveness, and toxicity. Therefore, it is necessary to have a strict quality control process, which is technically demanding (321).

Nanomedicines’ novelty and complexity create certain challenges for regulatory authorities such as the EMA or FDA. Unclear regulations and criteria associated with nanomedicines usually lead to confusion on the side of developers. A thorough toxicological investigation of the substance is required to assess the safety and toxicity of both drugs themselves and nanoparticles acting as a delivery vehicle for these drugs. Such evaluation should address chronic toxicity, bioaccumulation in different organs, in particular in the liver and spleen, and prognosis concerning the disease progression and development of complications after using a nanomedicine. Immunogenicity of the nanocarrier poses another safety hazard because the formation of protein coronas – proteins that stick to nanoparticle surfaces once the latter enter the body – might affect the targeting properties and biological characteristics of the nanocarrier, which makes it difficult to predict its further safety and efficiency. Overcoming these challenges would require intensive cooperation among the scientists, industry players, and regulatory authorities in devising standards in characterization and safety assessment techniques in order to make clinical translation of nanomedicines feasible (322).

**Table 1** demonstrates key distinctions in biodegradability, prolonged biodistribution, and regulatory requirements concerning organic versus inorganic nanocarriers. This difference is very important when trying to foresee the translational challenges and design nanomedicines which can be successfully applied in clinical practice.

**Table 1:** Regulatory and biodistribution considerations for biodegradable and non-biodegradable nanocarriers.

Aspect	Biodegradable nanocarriers	Non-biodegradable/inorganic nanocarriers	Ref
Typical materials	Lipids, PLGA, chitosan, PEGylated polymers	Gold nanoparticles, mesoporous silica, iron oxide, carbon nanotubes, graphene	(323) (324)
Degradability	Enzymatic or hydrolytic degradation into small, excretable molecules	Minimal or no physiological degradation; structural persistence	(325)
Primary clearance Pathways	Renal and biliary excretion after degradation	Reticuloendothelial system (liver, spleen), macrophage sequestration	(326)
Long-term biodistribution	Transient tissue presence; low long-term accumulation	Prolonged retention in liver, spleen, lymph nodes, sometimes lungs or bone marrow	(327)
Chronic accumulation risk	Low	Moderate to high, especially with repeated dosing	(328)
Protein corona concerns	Mainly affects circulation time and targeting efficiency	Dynamic corona evolution may alter biodistribution and immunogenicity over time	(280)
Immunogenicity profile	Generally low; well-characterized excipients	Potential chronic immune activation, complement engagement, granuloma formation	(326)
Regulatory classification	Often regulated as drug products or drug excipients	Frequently regulated as combination products (drug + device)	(306)
Regulatory pathway complexity	Relatively established and streamlined	More complex, case-by-case evaluation	(326)
Required toxicity studies	Acute and sub-chronic (28–90 days)	Acute, sub-chronic, and long-term (6–12 months or longer)	(326)
Biodistribution studies	Standard PK and tissue distribution	Mandatory quantitative long-term organ tracking (e.g., ICP-MS, imaging)	(325)
Dose accumulation assessment	Usually not required beyond standard PK	Required to assess cumulative exposure and saturation effects	(329)
Organ-specific safety focus	Liver and kidney metabolism	Liver, spleen, lungs, lymph nodes, sometimes brain	(330)
Impact of particle size/shape	Moderate	Critical determinant of biodistribution and toxicity	(331)
Manufacturing sensitivity	Moderate variability tolerated	Extremely sensitive to size, shape, surface chemistry	(326)
Imaging/tracking requirements	Optional	Often mandatory for regulatory approval	(323)
Clinical translation history	Multiple FDA-approved products	Limited approvals; mostly diagnostic or localized applications	(332)
Advantages	Predictable clearance, lower long-term risk	High stability, multifunctionality (imaging, thermal, radiosensitization)	(325, 326, 329)
Relevance to repeated dosing	Suitable	Requires strict cumulative exposure justification	(329)

### 9.3 The future of nanomedicine in cancer treatment

The future of nanomedicine in the treatment of cancer rapidly moves towards personalizing cancer treatments, from universal approaches to those tailored for individuals. In future, the application of nanocarriers in cancer therapies will involve the use of carriers personalized to the patient’s biology (333).The future cancer treatment will incorporate advanced nanocarriers that will carry the drug directly to the site of action.

In future, the development of theranostic nanoplatforms will be an important step. Theranostic nanoparticles integrate both diagnostic imaging agents and the therapeutic agents into one nanoparticle carrier system. This approach will make it possible for clinicians to monitor and confirm that nanoparticles accumulate in the targeted site of treatment, identify patients that are likely to show an adverse response to the medication, and measure the efficacy of the treatment process (334).

But nanomedicine’s future will also entail its synergy with other therapeutic approaches as well. We will be able to develop nanocarriers designed to act in concert with immunotherapies aimed at converting the immunosuppressive TME into an “activating” one where “cold” tumors would become “hot” and amenable to treatment using checkpoint inhibitors (335). Alternatively, nanomaterials may also be employed to deliver gene editing equipment like CRISPR/Cas9 system to mutate specific genes and inhibit the cancer cells’ ability to develop a resistance to treatment (336).Machine learning and AI have an essential part to play in this implementation process. In particular, the use of AI allows for rapid analysis of existing material to model the behavior of nanoparticles prior to synthesis. Machine learning can also help in developing personalized nanocarrier systems tailored specifically to the needs of each individual patient. The next phase in the evolution of nanomedicines will involve smart materials, combinatorial treatments, and intelligent computer design (285, 337).

## 10 Conclusion

The tumor microenvironment poses as one of the key challenges in cancer treatment through a range of biological and physical barriers that significantly inhibit the effectiveness of therapy. In general, the peculiarities of the tumor microenvironment including anomalous vasculature, dense extracellular matrix, high interstitial fluid pressure, hypoxia, and strong immunosuppression make it difficult for drugs to penetrate the tumor and promote its growth and progression.

This paper reviews the ways in which nanotechnology provides a valuable and versatile means of combating all the listed barriers to drug delivery in the tumor. Nanocarriers can improve drug distribution in the tumor through enhanced passive drug delivery using the EPR effect as well as targeting techniques that include active transport into the tumor environment. More advanced “smart” drug carriers that respond to specific triggers within the tumor microenvironment, such as changes in pH or enzyme activity, can ensure controlled drug delivery inside the tumor with an optimal level of toxicity. Most importantly, nanotherapy enables the active modulation of the tumor microenvironment by breaking down the ECM, normalizing the vasculature, reducing hypoxia, and reversing immunosuppression.

The future of TME-targeted nanomedicine is about its transformation into an active element of a complete personalized therapy for cancer patients. The idea is to go beyond any changes to make steps towards a new era of oncology, in which every treatment method will be determined by unique properties of each tumor’s TME. As for future nano-delivery systems, these will be multifunctional multi-stage devices created with the help of AI and being able to perform sequentially programmed operations. This system should not only carry combined therapeutic drugs, for example, chemotherapy, gene therapy, and immunotherapy, but also monitor their impact on the TME in order to provide a real-time assessment. Solving the problem of large-scale manufacturing and overcoming the difficulties related to regulation are the key points on the way to the implementation of this concept. Nevertheless, with the achievement of this goal, the future of TME-targeted nanomedicine will mean cancer therapy for every individual patient.
